# The dominant role of proofreading exonuclease activity of replicative polymerase ε in cellular tolerance to cytarabine (Ara-C)

**DOI:** 10.18632/oncotarget.16508

**Published:** 2017-03-23

**Authors:** Masataka Tsuda, Kazuhiro Terada, Masato Ooka, Koji Kobayashi, Hiroyuki Sasanuma, Ryo Fujisawa, Toshiki Tsurimoto, Junpei Yamamoto, Shigenori Iwai, Kei Kadoda, Remi Akagawa, Shar-Yin Naomi Huang, Yves Pommier, Julian E. Sale, Shunichi Takeda, Kouji Hirota

**Affiliations:** ^1^ Department of Radiation Genetics, Graduate School of Medicine, Kyoto University, Yoshidakonoe, Sakyo-Ku, Kyoto 606-8501, Japan; ^2^ Department of Chemistry, Graduate School of Science and Engineering, Tokyo Metropolitan University, Hachioji-Shi, Tokyo 192-0397, Japan; ^3^ Department of Biology, School of Sciences, Kyushu University, Nishi-Ku, Fukuoka 819-0395, Japan; ^4^ Division of Chemistry, Graduate School of Engineering Science, Osaka University, Toyonaka, Osaka 560-8531, Japan; ^5^ Division of Radiation Life Science, Research Reactor Institute, Kyoto University, Kumatori, Sennan, Osaka 590-0494, Japan; ^6^ Developmental Therapeutics Branch and Laboratory of Molecular Pharmacology, Center for Cancer Research, National Cancer Institute, National Institutes of Health, Bethesda, MD 20892, USA; ^7^ Medical Research Council Laboratory of Molecular Biology, Cambridge, CB2 0QH, UK

**Keywords:** replicative polymerase ε, proofreading exonuclease, chainterminator, nucleoside analog, cytarabine (Ara-C)

## Abstract

Chemotherapeutic nucleoside analogs, such as Ara-C, 5-Fluorouracil (5-FU) and Trifluridine (FTD), are frequently incorporated into DNA by the replicative DNA polymerases. However, it remains unclear how this incorporation kills cycling cells. There are two possibilities: Nucleoside analog triphosphates inhibit the replicative DNA polymerases, and/or nucleotide analogs mis-incorporated into genomic DNA interfere with the next round of DNA synthesis as replicative DNA polymerases recognize them as template DNA lesions, arresting synthesis. To address the first possibility, we selectively disrupted the proofreading exonuclease activity of DNA polymerase ε (Polε), the leading-strand replicative polymerase in avian DT40 and human TK6 cell lines. To address the second, we disrupted *RAD18*, a gene involved in translesion DNA synthesis, a mechanism that relieves stalled replication. Strikingly, *POLE1^exo−/−^* cells, but not *RAD18^−/−^* cells, were hypersensitive to Ara-C, while *RAD18^−/−^* cells were hypersensitive to FTD. gH2AX focus formation following a pulse of Ara-C was immediate and did not progress into the next round of replication, while gH2AX focus formation following a pulse of 5-FU and FTD was delayed to the next round of replication. Biochemical studies indicate that human proofreading-deficient Polε-exo^−^ holoenzyme incorporates Ara-CTP, but subsequently extend from this base several times less efficiently than from intact nucleotides. Together our results suggest that Ara-C acts by blocking extension of the nascent DNA strand and is counteracted by the proofreading activity of Polε, while 5-FU and FTD are efficiently incorporated but act as replication fork blocks in the subsequent S phase, which is counteracted by translesion synthesis.

## INTRODUCTION

Nucleoside analogs have been widely used for treating cancer and viral infections. Three anti-cancer chemotherapeutic drugs, Cytosine arabinoside (Ara-C, cytarabine), 5-Fluorouracil (5-FU) and Trifluridine (FTD), are efficiently incorporated into genomic DNA during DNA replication [1, 2]. However, the molecular mechanism of the cytotoxic effect of these drugs remains uncertain. In particular, it is unclear to what extent these nucleoside analogs interfere with DNA replication at the point of their misincorporation and/or whether they subsequently interfere with DNA synthesis by acting as blocks on the DNA template in the subsequent S phase. The inhibitory effect of Ara-CTP on purified replicative DNA polymerases has been reported for Polα, which is involved in priming DNA synthesis and lacks proofreading activity, but not proofreading-proficient Polδ or Polε [3, 4], polymerases thought to be responsible for lagging and leading strands synthesis, respectively [5]. Paradoxically, Ara-C slows down DNA synthesis *in vivo* suggesting inhibition of DNA polymerization, while a large amount of Ara-CMP is eventually incorporated into genomic DNA [6–8]. Incorporated Ara-CMP might locally alter the DNA structure [9], and would be expected to block the progression of DNA replication forks at the Ara-CMP site on template strands. Translesion DNA synthesis (TLS) and homologous recombination (HR) alleviate such replication blockage [10–12]. Although the above mechanisms could all explain cellular sensitivity to Ara-C, and other nucleoside analogs, no studies have actually measured the contribution of the individual DNA damage repair and tolerance pathways to cellular resistance to nucleotide analogs.

The anti-viral nucleoside analogs, abacavir (ABC), azidothymidine (AZT, zidovudine) and lamivudine, are imported by cells, phosphorylated, and incorporated by viral DNA polymerases. These three agents are known as chain-terminating-nucleoside-analog (CTNA), as their incorporation inhibits further extension due to their lack of 3′ hydroxyl group (3′-OH), leading to premature termination of viral genome synthesis [13, 14]. Biochemical studies using the catalytic subunits of Polδ and Polε have indicated that anti-viral CTNAs are incorporated by viral DNA/RNA polymerases considerably more efficiently than by the replicative DNA polymerases of host cells [15, 16]. Nonetheless, substantial quantities of anti-viral CTNAs are likely to be mis-incorporated into genomic DNA of the host by Polδ and Polε, considering that human genome is about five orders of magnitude larger than the average size of the retrovirus genome. In fact, ABC has been used for treating adult T cell leukemia (ATL), since ATL cells are unable to efficiently eliminate mis-incorporated ABC from the 3′ end of primers due to a defect in tyrosyl-DNA phosphodiesterase 1 (TDP1) [17]. An unsolved question is whether the proofreading activity of the replicative DNA polymerases are capable of efficiently eliminating nucleotide analogs as efficiently as it eliminates mis-incorporated dNTPs.

Mammalian Polε holoenzyme consists of four subunits, p261, p59, p17 and p12, with the p261 subunit containing both the DNA polymerase and proofreading 3′ to 5′ exonuclease domains [18–20]. Mice deficient in the proofreading activity of Polε and Polδ show enhanced mutagenesis and carcinogenesis [21–23]. However, no previous studies have measured the contribution of the proofreading activity to cellular resistance to nucleoside analogs. Stalling of Polε may have a stronger impact on the progression of replication forks than stalling of Polδ, as stalling of lagging-strand synthesis would leave single-strand gaps behind replication forks without interfering with their progression.

Exploiting isogenic mutants of chicken DT40 and human TK6 cell lines, we here report that we are able to temporally separate the killing effects of different nucleoside analogs by comparing the effects of the *POLE1^exo−/−^* mutant, which will loose the ability to remove incorporated nucleotide analogs from the elongating chain, and mutants in components of DNA damage tolerance and homologous recombination which mutants are impaired in the ability to alleviate replication forks blocked at template DNA lesions. We demonstrate that the proofreading exonuclease activity of Polε, but not damage tolerance or recombination pathways, critically contribute to cellular tolerance of Ara-C. In sharp contrast, 5-FU and FTD interfere with DNA replication when they are present on template strands resulting in replication fork collapse that is prevented by DNA damage tolerance and recombination pathways. The panel of the isogenic mutant clones we have employed here is likely to prove extremely useful for dissecting the cytotoxic mechanisms of novel chemotherapeutic nucleotide analogs on DNA replication.

## RESULTS

### Polε proofreading exonuclease deficient chicken DT40 mutant cells exhibit hypersensitivity to Ara-C

**Figure 1:**
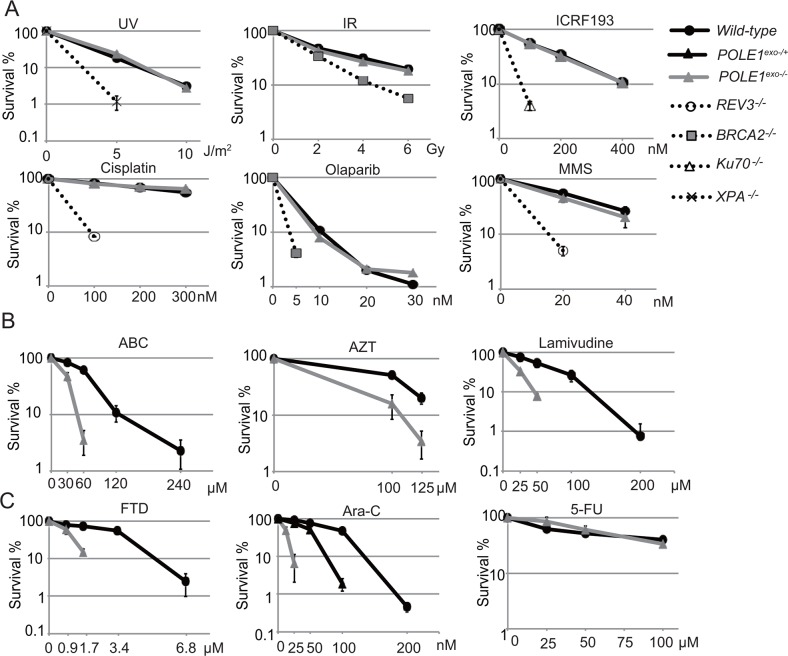
Important role of Polε exonuclease for cellular tolerance to nucleoside analogs in DT40 cells **(A)** Liquid-culture cell survival in the presence of the indicated genotoxic agents. The dose is displayed on the x-axis on a linear scale, while the percentage fraction of surviving cells is displayed on the y-axis on a logarithmic scale. Error bars show the SD of mean for three independent assays. **(B** and **C)** Survival curve of cells treated with the indicated nucleoside analogs. The sensitivity of cells to these nucleoside analogs was measured with methylcellulose colony formation assay [41]. Clinically relevant concentrations are 0.1 to 10 μM for ABC, AZT and Lamivudine, 100 nM for FTD, 30 nM for Ara-C and 10 μM for 5-FU [1, 32, 55].

To analyze the role of the proofreading exonuclease activity of Polε, we inactivated the exonuclease by inserting point mutations into the *POLE1* gene encoding the p261 subunit of Polε in DT40 cells (Supplementary Figure 1A-1B). We verified successful insertion of the mutations by RT-PCR and nucleotide sequencing (Supplementary Figure 1C). The resulting *POLE1^exo−/−^* cells proliferated slightly slower than *wild-type* cells and exhibited an increase in the fraction of sub-G1 dead cells (Supplementary Figure 1D-1E). We measured sensitivity to exogenous DNA damaging agents. *POLE1^exo−/−^* DT40 cells were not sensitive to cisplatin, UV, ICRF193 (Topoisomerase 2 catalytic inhibitor), γ-rays (ionizing-radiation (IR)), or olaparib (poly[ADP-ribose]polymerase inhibitor) (Figure 1A). However, *POLE1^exo−/−^* cells were more sensitive to ABC, AZT and lamivudine than *wild-type* cells (Figure 1B), indicating an important role of the exonuclease activity of Polε in suppressing the toxic effects of these anti-viral agents. Moreover, *POLE1^exo−/−^* DT40 cells were about 6-fold more sensitive to Ara-C, as judged from an inhibition concentration 50% (IC_50_), revealing that the exonuclease activity plays a key role in cellular tolerance to Ara-C (Figure 1C). The heterozygous mutant (*POLE1^exo-/+^*) was also sensitive to Ara-C (Figure 1C). These observations suggest that the exonuclease of Polε might eliminate Ara-CMP immediately after mis-incorporation by Polε and that this mis-incorporation causes cytotoxicity. *POLE1^exo−/−^* cells were also sensitive to FTD, but not to 5-FU. These observations support the notion that the cytotoxicity of Ara-C and FTD is attributable to replication stress caused by incorporation of these nucleotide analogs by DNA polymerases.

### The human Polε holoenzyme incorporates Ara-CTP and dCTP with the same efficiency

To further examine the role played by proofreading exonuclease activity of Polε in the removal of nucleotide analogs, we purified the intact human Polε holoenzyme (Polε (WT)) and exonuclease-deficient holoenzyme (Polε (exo-)) [24]. Polε (WT) and Polε (exo-) were expressed and purified with the same efficiency (Supplementary Figure 2A), indicating that the absence of the exonuclease activity does not diminish the stability of the other three components of the holoenzyme. Polε (exo-) did not induce detectable DNA degradation even in the absence of dNTP, while the lack of dNTP normally strongly stimulates the exonuclease activity in Polε (WT) (Supplementary Figure 2B, 2C). We therefore conclude that the D275A mutation completely abolishes the exonuclease activity of Polε.

**Figure 2:**
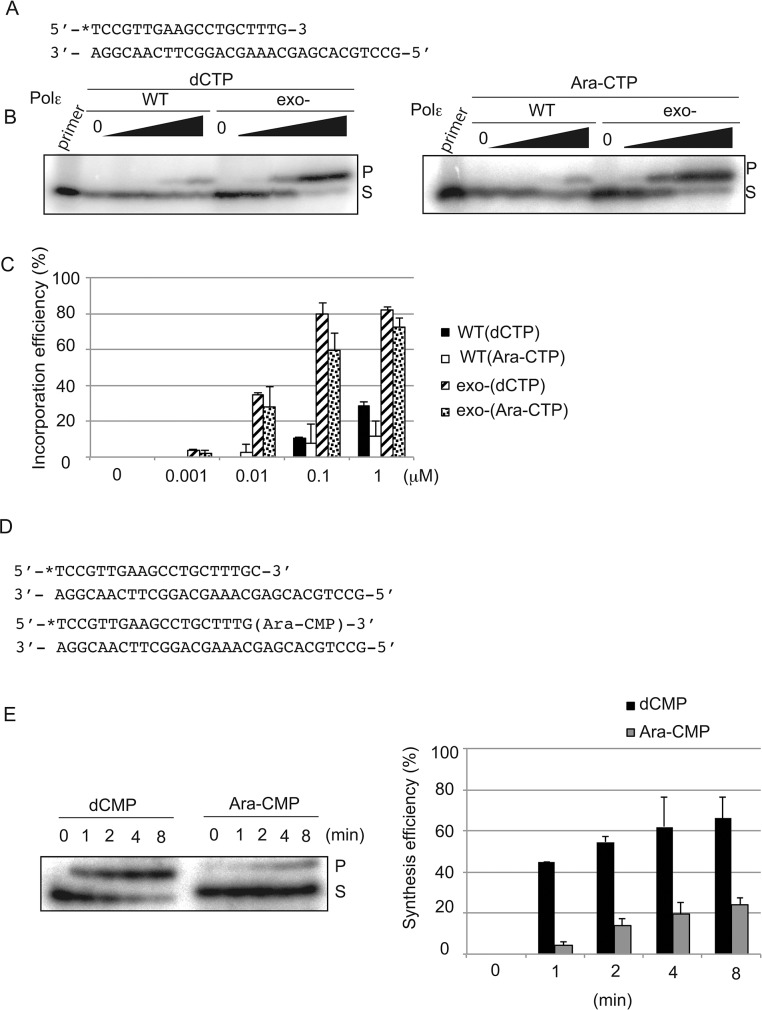
Human Polε holoenzyme efficiently incorporates Ara-CTP but further extend poorly *in vitro* **(A)** The nucleotide sequence of oligonucleotide primers and templates used for the experiment of **(B)**. The position of radiolabel with ^32^P is noted with asterisk. **(B)** A single nucleotide addition to the 3′ end of the primer by Polε (WT) and Polε (exo-) with varying concentrations of the indicated nucleotide analogs. The actual concentrations are shown in **(C)**. Reaction was carried out with 40 nM Polε and 8 nM of the primer/template strands in the absence of dNTPs for 15 min. The substrate ‘S’ represents the primer, and ‘P’ represents products of a single nucleotide incorporation, either dCMP or Ara-CMP. **(C)** Quantification of the single nucleotide incorporation efficiency. The histogram shows the relative yield of products at indicated concentration of the dCTP or Ara-CTP. The incorporation efficiency indicates the ratio of the amount of the elongated product relative to that of the unextended primer. Error bars show the SD for three independent assays. **(D)** Sequences of oligonucleotide primers and templates used for the experiment of **(E)**. The 3′ end of the primers is labeled with ^32^P, and the 3′ end carries either dCMP or Ara-CMP. **(E)** A single nucleotide extension was analyzed using 40 nM of Polε (exo-) and 8 nM of ^32^P labeled primers carrying dCMP or Ara-CMP at the 3′ end in the presence of 10 μM dTTP for the indicated duration. The percentage of products relative to the input primer is plotted with time as mean ±SD of three independent experiments.

To examine the incorporation of nucleotide analogs by the Polε (exo-) holoenzyme at the 3′ end of primers, we used the 30-mer template and 19-mer primer DNA strands that allow the incorporation of a single nucleotide analog, but not more, on the 3′ end of primer (Figure 2A). We examined the incorporation of deoxycytidine triphosphate (dCTP), Ara-CTP (Figure 2B and 2C), carbovir triphosphate (the active form of ABC [25]), and lamivudine triphosphate (Supplementary Figure 3). Surprisingly, Polε (exo-) incorporated Ara-CTP and dCTP with similar efficiency, while it incorporated carbovir and lamivudine triphosphate with lower efficiency by one and three orders of magnitude respectively in comparison to dCTP (Figure 2B and 2C, and Supplementary Figure 3A–3C). Thus, Polε (exo-) does not distinguish Ara-CTP from intact dNTPs as a substrate *in vitro*.

To analyze the role of the exonuclease activity, we measured primer extension by the Polε (WT) holoenzyme. Polε (WT) and Polε (exo-) incorporated dCTP into ~20% of primers in the presence of 1μM and 0.01μM of dCTP, respectively (Figure 2B and 2C). Thus, the incorporation efficiency of dCTP by Polε (WT) was a few orders of magnitude lower than that by Polε (exo-), indicating that the proofreading exonuclease activity very efficiently eliminates incorporated dCMP. Surprisingly, Polε (WT) incorporated Ara-CTP and dCTP with very similar efficiency. Likewise, Polε (exo-) incorporated Ara-CTP and dCTP with very similar efficiency. These observations indicate that the balance between the incorporation and elimination by Polε (WT) is similar for Ara-CTP and dCTP. Thus, the proofreading activity of Polε (WT) may not be able to distinguish incorporated Ara-CMP from dCMP. In contrast with Ara-CTP, at least ten and 10^4^ times higher concentrations of carbovir and lamivudine triphosphate, respectively, than dCTP were required to yield a products equivalent to 10% of the total amount of the primer (Figure 2B and 2C and Supplementary Figure 3A–3C). We conclude that Ara-CTP has a unique characteristic in the sense that Polε incorporates it as efficiently as dCTP and that the proofreading activity eliminates mis-incorporated Ara-CMP with very similar efficiency as eliminating incorporated dCMP. The data suggests that the exonuclease may excise mis-incorporated Ara-CMP as a consequence of its premature chain termination activity rather than recognizing mis-incorporated Ara-CMP as a mispair.

### The human Polε holoenzyme is capable of extending DNA synthesis from incorporated Ara-CMP

We then investigated whether Ara-CMP incorporated at 3′ end of newly synthesized strand indeed blocks extension of the nascent DNA synthesis. To this end, we prepared a primer carrying Ara-CMP at its 3′ end (Figure 2D). We also prepared a primer carrying dCMP at its 3′ end for a control experiment (Figure 2D). We prepared template strands, where only a single dTTP is incorporated next to the Ara-CMP and dCMP in the primer. Polε (exo-) efficiently extended from the intact primer carrying dCMP at its 3′ end and over 40% of primer incorporated dTMP within one-minute incubation (Figure 2E and 2F). By contrast, Polε (exo-) extended less efficiently and only 20% of primer carrying Ara-CMP at its 3′ end incorporated dTMP even after 8 min. Nonetheless, Polε (exo-) retains the capability of maintaining DNA synthesis from incorporated Ara-CMP. These biochemical data agree with the *in vivo* observation that Ara-C interferes with DNA replication to some extent but is also frequently incorporated into genomic DNA [6–8]. In summary, Ara-CTP is incorporated by Polε with the same efficiency as dCTP but then partially inhibits extension from the Ara-CMP at the 3′ primer terminus.

### The exonuclease activity of Polε facilitates DNA synthesis in the presence of Ara-C *in vitro*

To test whether the proofreading 3′ to 5′ exonuclease activity of Polε can eliminate nucleotide analogs, we set up an *in vitro* assay using primers containing nucleotide analogs (Supplementary Figure 4A). Firstly, we assessed the effect of free dNTP on the exonuclease activity. In general, increasing the dNTP concentration stimulates DNA synthesis activity and suppresses the exonuclease activity [26]. However, in the case of Polε the exonuclease activity was not suppressed even by a physiological concentration (10 μM) of dNTP (Supplementary Figure 2C), indicating that the Polε (WT) holoenzyme possesses an extremely strong intrinsic exonuclease activity. The absence of CMG helicase and PCNA in this *in vitro* experiment may cause an apparent shift from DNA synthesis to degradation by the exonuclease activity [27–30]. Polε (WT), but not Polε (exo-), exhibited efficient removal of 3′ Ara-CMP, lamivudine monophosphate and carbovir monophosphate (Supplementary Figure 4).

**Figure 3:**
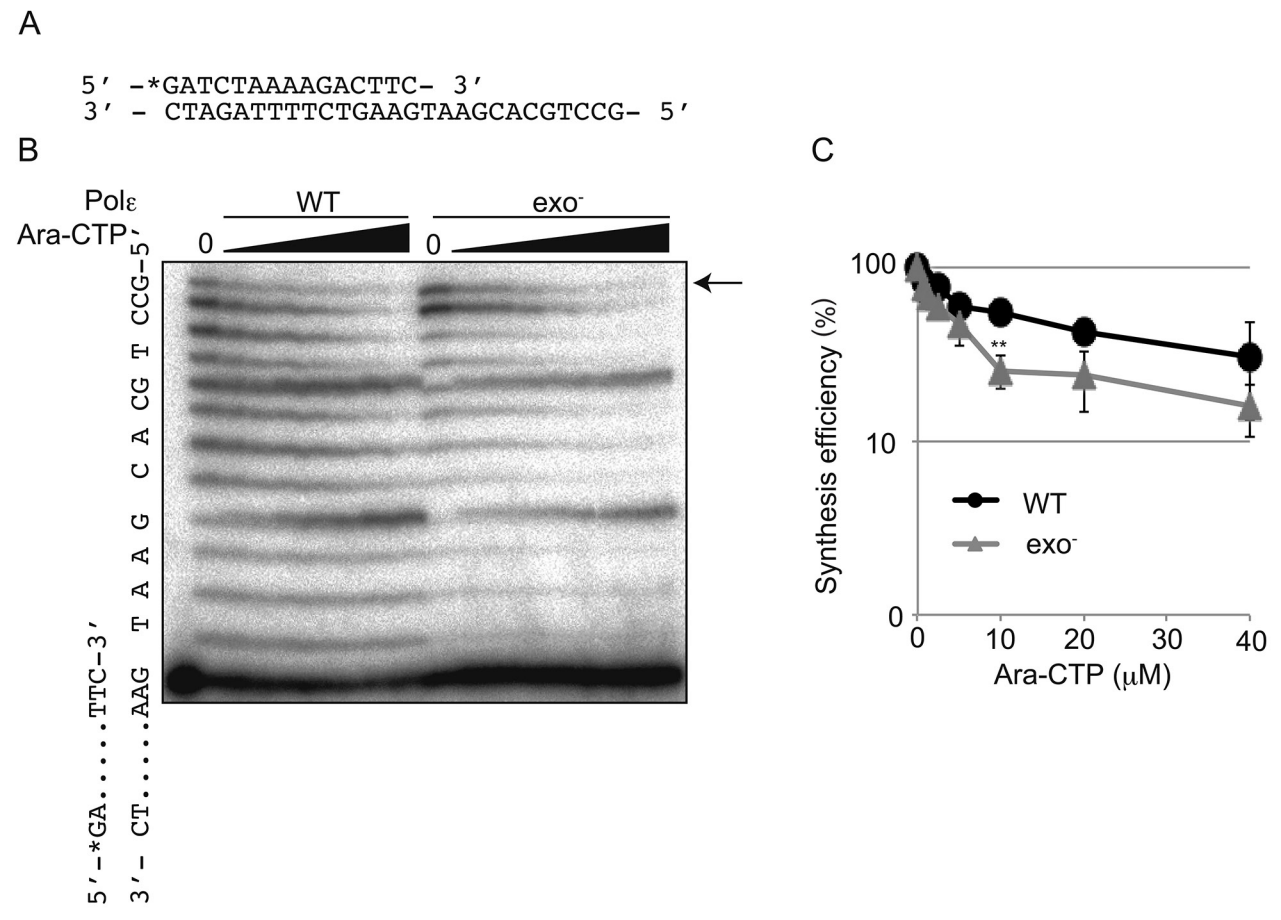
Extension by Polε in the presence of Ara-CTP and 10 μM dNTP **(A)** The sequences of oligonucleotide primers and templates. The position of radiolabel with ^32^P is noted with asterisk. **(B)** Gel image showing DNA synthesis by the Polε (WT) and Polε (exo-) holoenzymes in the presence of indicated concentrations of Ara-CTP. The position of the fully elongated products is indicated with an arrow. **(C)** The relative yield of fully elongated products is plotted against increasing Ara-CTP concentrations as mean ±SD of three independent experiments. Asterisks indicate statistical significance. **P=0.0020.

Such efficient removal led us to test whether the exonuclease activity of Polε facilitates DNA synthesis in the presence of Ara-CTP. To this end, we assessed the effect of increasing concentrations of Ara-CTP on DNA synthesis by Polε (WT) as well as Polε (exo-) in the presence of 10 μM dNTP using the same template and primer strands (Figure 3A) as those shown in Supplementary Figure 2C. Note that ~40 % of the primer was shortened by the exonuclease activity of Polε (WT) in the presence of 10 μM dNTP. We found that even in the presence of this substantial degradation by Polε (WT), the amount of the product fully replicated by Polε (WT) was higher than that by Polε (exo-) (Figure 3B and 3C), as 10 μM Ara-CTP suppressed Polε (WT) dependent DNA synthesis by 45% and Polε (exo-) dependent one by 75%. This observation highlights the critical role for the exonuclease activity of Polε in maintenance of DNA replication by Polε when Ara-CTP is present.

### The dominant role for the Polε proofreading activity in cellular tolerance to Ara-C

**Figure 4:**
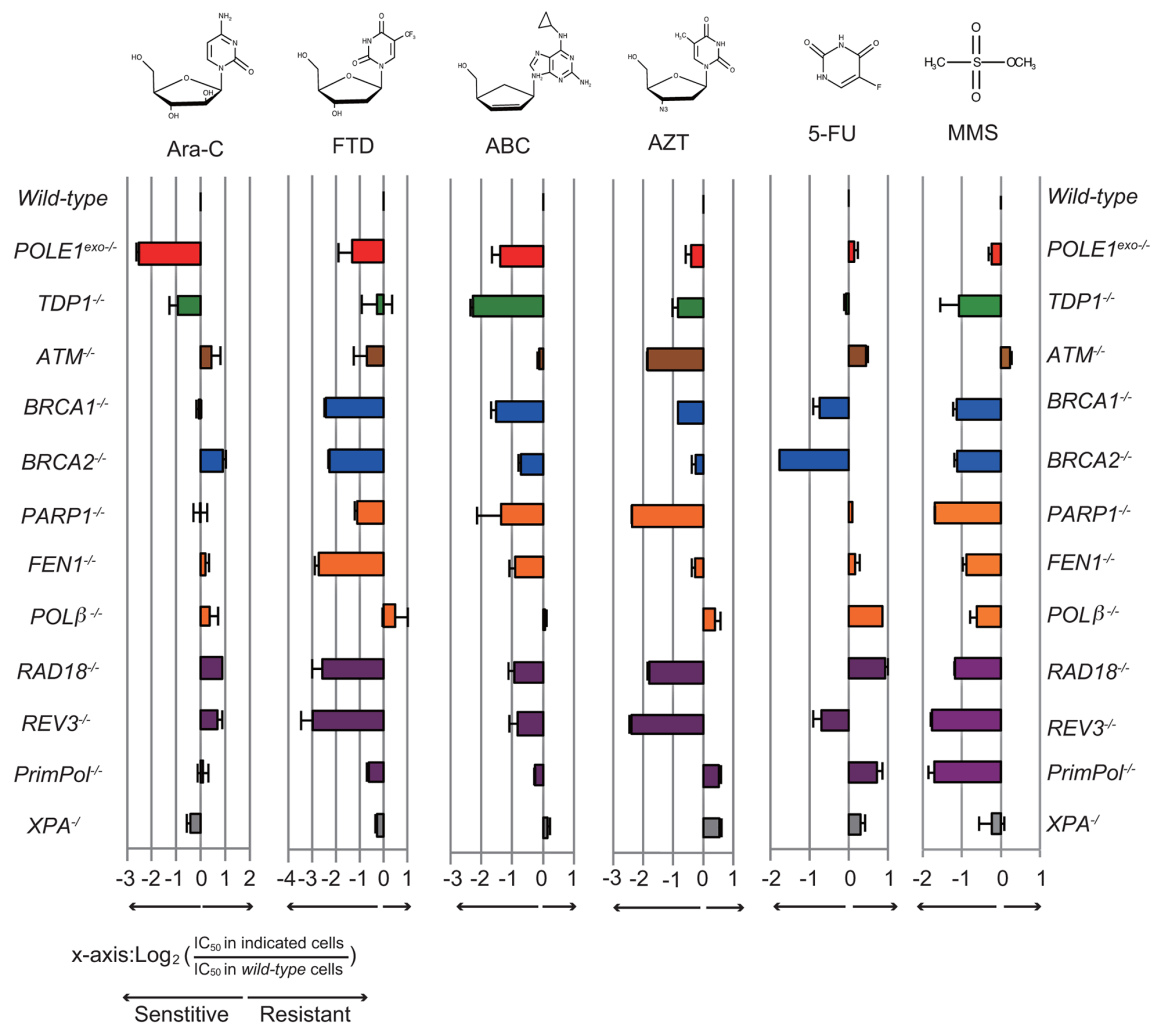
Sensitivity profiles of the indicated nucleoside analogs in the selected DNA repair deficient DT40 cells The colony survival was measured as in Figure 1. The relative sensitivity of each isogenic mutant DT40 cells compared to *wild-type* DT40 cells was scored as log_2_ (IC_50_ in indicated mutant cells)/(IC_50_ in *wild-type* cells). Negative (left) or positive (right) scores indicate that the cell line was either sensitive or resistant to the specified nucleoside-analog, respectively. Each bar is colored according to the category of DNA repair function: red, *POLE1^exo−/−^*; green, removal of Top1 cleavage complex; brown, checkpoint; blue, HR; orange, base excision repair; gray, nucleotide excision repair, and purple, postreplication repair. Error bars show the SD of the mean for three independent assays.

An important question is how Ara-C kills *wild-type* cells proficient in the exonuclease activity of Polε, given this activity contributes significantly to the maintenance of DNA replication. There are two possible and not mutually exclusive mechanisms. First, Ara-C causes replication stress by partially inhibiting the extension of DNA synthesis upon its incorporation even in the presence of intact exonuclease activity. The second possibility, given Ara-CMP is very frequently mis-incorporated into the genomic DNA [6–8], mis-incorporated Ara-C interferes with the next round of DNA synthesis. To address the second mechanism, we measured the colony survival of isogenic DT40 mutants (Table 1), including *POLE1^exo−/−^* cells, TLS-deficient mutants (*RAD18^−/−^* and *REV3^−/−^*), HR-deficient mutants (*BRCA1^−/−^* and *BRCA2^−/−^*) as well as mutants of base excision repair (*PARP1^−/−^*, *FEN1^−/−^* and *POLβ^−/−^*), to nucleoside analogs and an alkylating agent, methyl methane sulfonate (MMS). We then calculated IC_50_, at which concentration the colony survival was decreased by half relative to untreated cells. Figure 4 shows the ratio of IC_50_ of individual isogenic mutants relative to IC_50_ of *wild-typ*e DT40 cells on a logarithmic scale. Supplementary Figure 5 shows actual colony survival of isogenic DT40 mutants. Remarkably, only *POLE1^exo−/−^* cells, but not the HR or TLS mutants, showed hypersensitivity to Ara-C (Figure 4). An important pathway for the elimination of nucleotide analogs is Tyrosyl-DNA phosphodiesterase 1 (TDP1). TDP1 is capable of eliminating nucleotide analogs from 3′ end of primers [17, 31] and also plays a role in cellular tolerance to Ara-C (Figure 4). This sensitivity profile of Ara-C is in stark contrast with that of FTD, which exhibited significantly higher cytotoxicity to both TLS and HR mutants in addition to *POLE1^exo−/−^* cells. These observations suggest that the cytotoxicity of Ara-C results from its partial chain terminating activity even in proofreading-proficient *wild-type* cells. Although Ara-CMP is actively incorporated during DNA replication [6, 7], Ara-CMP included in the genomic DNA has a very limited impact on the second round of replication.

**Table 1:** List of cell lines used in this study

Genotype	Name of cell line and species	Marker genes	Function of deleted genes	Reference
*POLE1^exo−/−^*	Chicken DT40	*BSR^R^*	Proofreading	This study
*TDP1^−/−^*	Chicken DT40	*HYG^R^, PURO^R^*	Removal of TOP1 cleavage complex	[45]
*ATM^−/−^*	Chicken DT40	*NEO^R^, PURO^R^*	Checkpoint control	[46]
*BRCA1^−/−^*	Chicken DT40	*PURO^R^, HIS^R^*	HR	[47]
*BRCA2^−/−^*	Chicken DT40	*HYG^R^*	HR	[47]
*PARP1^−/−^*	Chicken DT40	*HIS^R^, BSR^R^*	BER	[48]
*Fen1^−/−^*	Chicken DT40	*HYG^R^, PURO^R^*	BER	[49]
*POLβ^−/−^*	Chicken DT40	*BSR^R^, HIS^R^*	BER	[50]
*RAD18^−/−^*	Chicken DT40	*HIS^R^, HYG^R^*	PRR	[51]
*REV3^−/−^*	Chicken DT40	*HIS^R^, BSR^R^*	TLS	[52]
*XPA^−/^*	Chicken DT40	*PURO^R^*	NER	[53, 54]
*PrimPol^−/−^*	Chicken DT40	*PURO^R^, BSR^R^*	Repriming of replication and TLS	[12]
***POLE1^exo−/−^***	Human TK6	*PURO^R^*	Proofreading	This study
*RAD18^−/−^*	Human TK6	*PURO^R^, HYG^R^*	PRR	This study

Like MMS, ABC sensitizes cells deficient in base excision repair, HR and TLS, as reported previously [17] (Figure 4). AZT also sensitizes isogenic mutants of the three pathways. Thus, these anti-viral CTNAs are somehow incorporated into the genomic DNA during DNA replication, incorporated nucleotide analogs on template strands significantly interfere with the subsequent round of DNA replication, and resulting stalled replication forks are released by HR and TLS. TDP1 plays a more important role in cellular tolerance to ABC than does the exonuclease activity of Polε, while the exonuclease activity is considerably more important in cellular tolerance to Ara-C. In summary, Ara-C is unique among the nucleoside analogs tested in the sense that its cytotoxicity depends exclusively on replication stress when it is incorporated by replicative DNA polymerases.

### The proofreading activity of human Polε is required for cellular tolerance to nucleoside analogs

**Figure 5:**
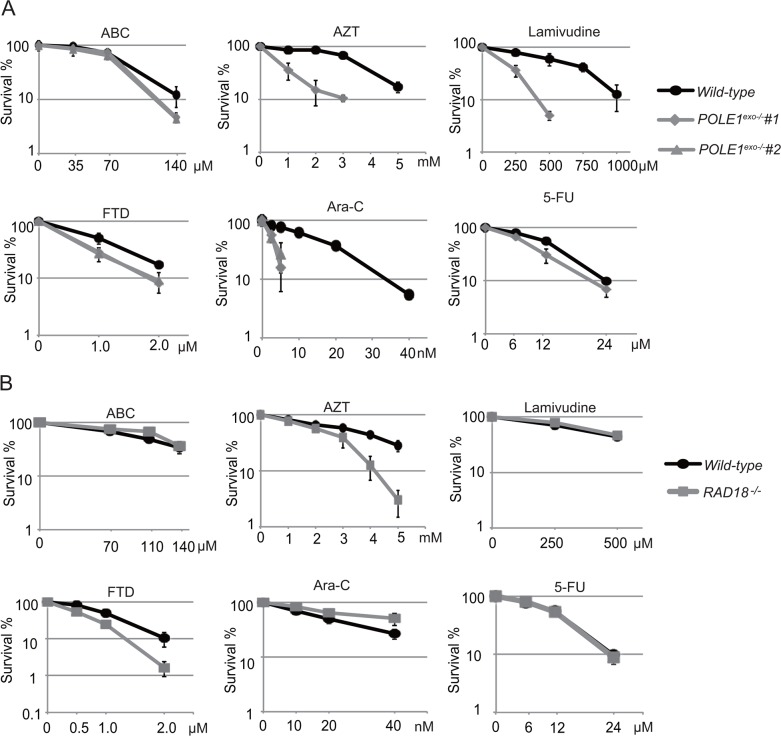
The important role of Polε exonuclease in cellular tolerance to nucleoside analogs in the human TK6 cell line **(A)** The sensitivity of *POLE1^exo−/−^* cells to the indicated nucleoside analogs. The colony survival was measured as in Figure 1. The numbers of surviving colonies relative to those of untreated controls are shown on the y-axis on a logarithmic scale, while the concentrations of the nucleoside analogs are displayed on the x-axis on a linear scale. Error bars show the SD of the mean for three independent assays. **(B)** The sensitivity of *RAD18^−/−^* TK6 cells to the indicated nucleoside analogs. Data are shown as in **(A)**.

To investigate the role of the Polε exonuclease activity in human cells, we generated a *POLE1^exo−/−^* mutant of the human TK6 B cell line (Supplementary Figure 6) and measured cellular sensitivity to nucleoside analogs (Figure 5A). We also tested *RAD18^−/−^* TK6 cells as a representative mutant of replication block tolerance pathway (Supplementary Figure 7 and Figure 5B). The human *POLE1^exo−/−^* mutant showed a sensitivity profile very similar to that of the chicken *POLE1^exo−/−^* mutant (compare Figure 1B, 1C and 5A). Of note, *POLE1^exo−/−^* TK6 cells were hypersensitive to Ara-C, and even the heterozygous mutant (*POLE1^exo-/+^*) was moderately sensitive to Ara-C (Supplementary Figure 8). We therefore conclude that Polε efficiently eliminates 3′ blocking Ara-CMP mis-incorporated by itself. The elimination greatly contributes to cellular tolerance to Ara-C in both human and chicken cells. *POLE1^exo−/−^* TK6 cells were also hypersensitive to AZT and lamivudine (Figure 5A). Thus, these nucleoside analogs are frequently incorporated by Polε, leading to premature termination of DNA replication in human cells. *RAD18^−/−^* TK6 cells (Figure 5B) showed a less pronounced phenotype compared with *RAD18^−/−^* DT40 cells (Figure 4). Nonetheless, the human and chicken *RAD18^−/−^* mutants displayed a similar overall sensitivity profile, including sensitivity to AZT and FTD but not to Ara-C.

### γH2AX focus formation following a pulse of Ara-C is immediate and not delayed to the next round of replication

**Figure 6:**
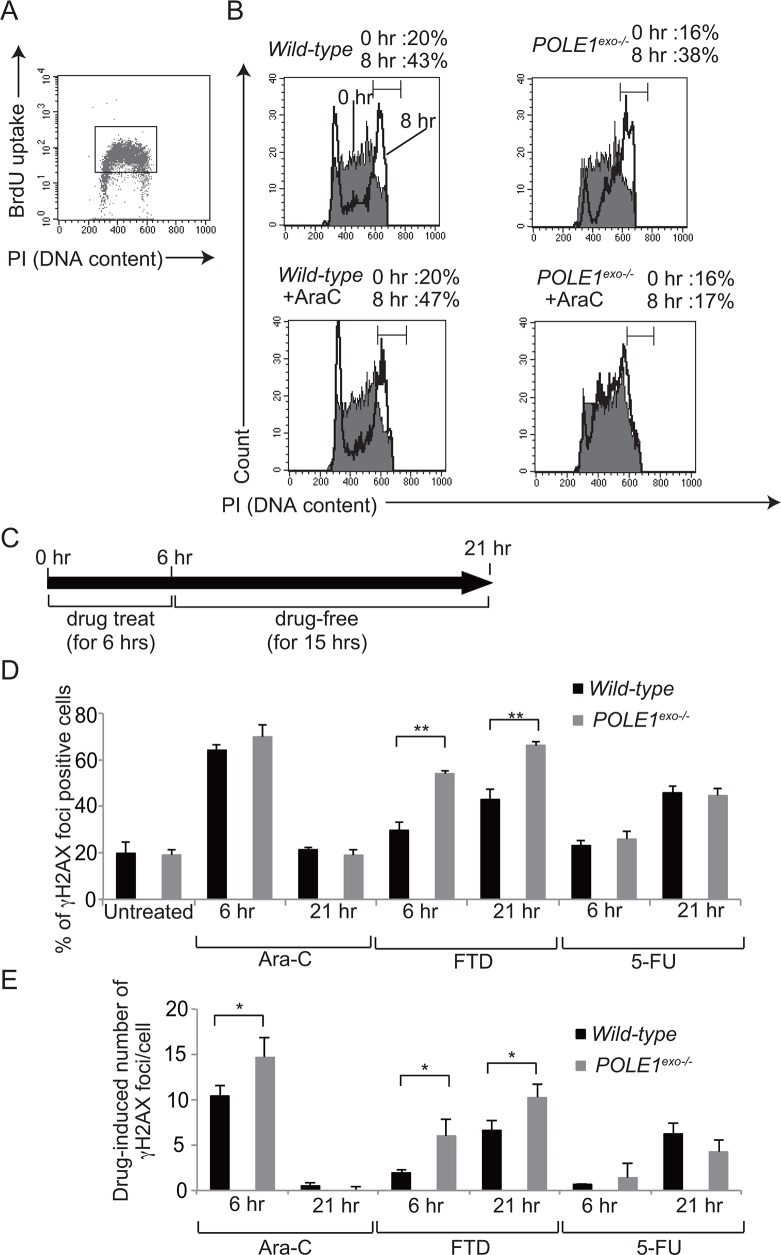
The effect of Ara-C on DNA replication and γH2AX focus formation **(A)** Representative cell-cycle distribution of the *wild-type* TK6 cells as measured by BrdU incorporation and DNA content in flow cytometric analysis. Cells were pulse-labeled with BrdU for 15 min, and subsequently stained with FITC-conjugated anti-BrdU to measure BrdU uptake (y-axis, log scale) and with propidium iodide to measure total DNA (y-axis, linear scale) in individual cells. Cells falling in the indicated gate are defined as the BrdU-positive cells being subjected to the pulse-chase analysis shown in B. **(B)** The BrdU-positive cells defined by the gate shown in A were chased with BrdU-free medium containing either zero (upper) or 30 nM Ara-C (lower) for eight hours. The filled and open histograms represent the DNA content of the BrdU-positive cells at 0 and 8 hours, respectively, after the BrdU pulse-labeling. The indicated bracket defines cells in the G2/M phase. The numbers shown on top indicate the percentage of the G2/M cells in the BrdU-positive cells at 0 and 8 hour chase periods. **(C)** The experimental protocol for the immunofluorescent visualization of subnuclear γH2AX focus formation in *wild-type* and *POLE1^exo−/−^* TK6 cells. Following pulse-treatment with either 30 nM Ara-C, 100 nM FTD, or 10 μM 5-FU for 6 hours, cells were incubated for 15 hours in drug-free medium. γH2AX foci were measured at 6 and 21 hours (**D** and **E**). The bar graph represents mean and SD of % γH2AX-foci positive cells (> seven foci per cell) **(D)** and the number of Ara-C-induced γH2AX **(E)** in three independent experiments. The number of Ara-C-induced γH2AX was calculated by subtracting the number of spontaneously arising γH2AX foci from that of γH2X foci in Ara-C-treated cells. At least fifty nuclei were scored in each case. Statistical significance (by Student's *t*-test) is as follows: *, *P*<0.05; **, *P*<0.01; n.s., not significant.

We examined the effect of Ara-C on the cell cycle progression by BrdU pulse-chase labeling (Figure 6A and 6B). We pulse labeled S-phase cells with BrdU, and monitored the progression of the labeled cells through the cell cycle over an eight hours chase period in the presence of 30 nM Ara-C, a concentration close to serum concentration seen in treated patients [32]. In agreement with our biochemical data (Figure 3), *POLE1^exo−/−^* TK6 cells, but not *wild-type* cells, showed a significant delay in the progression from the S to G2/M phases during the chase period when Ara-C was present (Figure 6A, 6B). Thus, the Polε exonuclease activity significantly contributes to the progression of the S phase when replicative DNA polymerases mis-incorpotate Ara-CTP.

An important question is what effects Ara-C has on DNA replication in proofreading-proficient *wild-type* cells. To address this question, we exposed *wild-type* TK6 cells to Ara-C for 6 hours, and chased in drug-free medium for 15 hours (Figure 6C). While the single cell cycle time of TK6 cells is ~13 hours, Ara-C treatment slightly slowed cell cycle progression and cell cycle time of Ara-C treated cells became ~15 hours (Supplementary Figure 9). γH2AX foci represent double-strand breaks and replication stalling [33]. The percentage of γH2AX-foci-positive cells was highest immediately after the pulse-exposure of cells to Ara-C, and decreased to the basal level at 21 hour (Figure 6D, Supplementary Figure 10). We therefore conclude that even if the proofreading of Polε is active, mis-incorporation of Ara-CTP by replicative polymerases causes significant replication stress immediately after mis-incorporation, leading to cell death (Figures 1C and 5A).

We next examined the effects of Ara-C on the DNA replication of *POLE1^exo−/−^* cells. We defined γH2AX-foci positive cells as cells displaying more than seven foci per cell (Figure 6D), since the number of spontaneously arising γH2AX-foci did not exceed seven foci per cell. We also showed actual number of Ara-C-induced γH2AX-foci per cell (Figure 6E), since % γH2AX-foci positive cells were saturated at 6 hour as virtually all Ara-C-treated S/G2 phase cells display more than seven foci. *Polε p261exo^−/−^* cells displayed a higher number of Ara-C-induced γH2AX foci immediately after Ara-C treatment in comparison with *wild-type* cells (Figure 6E). The number of γH2AX-foci per cell returned to a background level in both *POLE1^exo−/−^* and *wild-type* cells at 21 hour, when the cells were undergoing the second round of the cell cycle after pulse-exposure to Ara-C (Supplementary Figure 9). Considering very frequent mis-incorporation of Ara-CMP into the genomic DNA [6–8], the data revealed that mis-incorporated Ara-C no longer cause replication stress during the second round of DNA replication, which agrees with no detectable sensitivity of TLS-deficient *RAD18^−/−^* cells to Ara-C (Figures 4 and 5B). In contrast with Ara-C, following pulse-exposure to FTD and 5-FU, the percentage of γH2AX-foci-positive cells was increased at 21 hour (Figure 6D and 6E). Pulse-exposure to FTD and 5-FU did not significantly affect cell cycle progression (Supplementary Figure 9). These observations indicate that the molecular mechanisms underlying the cytotoxicity of Ara-C are distinctly different from that of FTD and 5-FU even when the proofreading activity of Polε is present.

To our surprise, TLS-deficient DT40 and human TK6 cells were hypersensitive to AZT (Figures 4, 5). These observations suggest the following hypothesis. A fraction of AZT may be incorporated into the genomic DNA despite its chain-terminating activity. During the second round of DNA replication, replicative DNA polymerases may stall at sites of AZT incorporated in the template strand, and TLS may restore the stalled replication forks. To test this hypothesis we measured γH2AX-foci after a pulse exposure of cells to AZT. The pulse-exposure did not significantly affect cell cycle progression (Supplementary Figure 11). Remarkably, the TLS-deficient *RAD18^−/−^* TK6 cells displayed more prominent γH2AX focus formation during the second round of DNA replication at 21 hour after the pulse-exposure in comparison with the first round of DNA replication, immediately after the 6 hour pulse exposure (Supplementary Figure 12). Taken together, a fraction of AZT can be incorporated by replicative DNA polymerases, and AZTs incorporated into genomic DNA strongly interfere with the next round of DNA replication leading to the formation of prominent γH2AX-foci.

The current data revealed differential effects of nucleoside analogs. Ara-C kills cycling cells by interfering with DNA replication during its incorporation by replicative DNA polymerases, while FTD and 5-FU interfere mainly with the subsequent round of DNA replication. AZT interferes with DNA replication not only as a chain-terminator but also during the subsequent round of DNA replication.

## DISCUSSION

**Figure 7:**
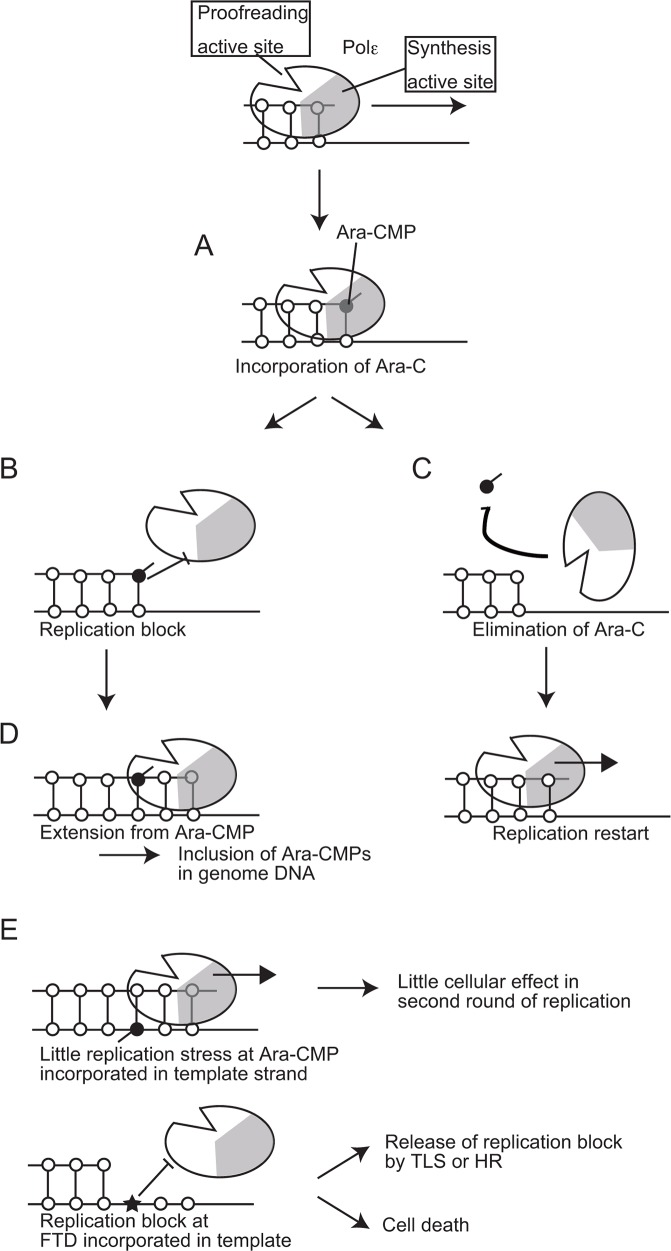
A model for the effects of Ara-CMP incorporated at 3′ ends of primers on DNA replication **(A)** Polε incorporates Ara-CTP with the same efficiency as does dCTP. **(B)** A significant delay in a huge number of the leading-strand replication causes strong replication stress leading to γH2AX focus formation. **(C)** More than 80% of the Ara-CMPs incorporated at the 3′ end of primers is eliminated by the proofreading activity of replicative DNA polymerases. **(D)** Extension from numerous residual Ara-CMPs results in them being included in the genomic DNA. **(E)** Incorporated Ara-CMP causes little effect in the second round of DNA replication, while FTD incorporated in genome causes replication block.

To examine the effects of clinically relevant nucleoside analogs on DNA replication, we created *POLE1^exo−/−^* cells and also prepared proofreading-deficient Polε holoenzyme. Here, we reveal that Ara-C, the first line chemotherapy agent for acute myeloid leukemia for the past 40 years is highly cytotoxic when incorporated by the replicative DNA polymerases, even in the presence of Polε exonuclease activity. In contrast, once incorporated into genomic DNA, Ara-CMP is no longer cytotoxic during the subsequent rounds of DNA replication (Figure 6D and 6E). We demonstrate that Polε exonuclease plays the dominant role in cellular tolerance to Ara-C (Figure 4 and 5A). In conclusion, Ara-C is a unique nucleoside analog in the sense that it induces significant replication stress at its incorporation by replicative DNA polymerases, while the large number of Ara-CMPs incorporated in the genomic DNA does not interfere with the subsequent round of DNA replication (Figure 7).

DNA replication is under significant stress in transformed cells [34]. It is a major target of anti-cancer chemotherapeutics including cisplatin, topoisomerase inhibitors, and nucleoside analogs such as ABC and Ara-C. The response to replication stress has been extensively studied by treating cells with hydroxyurea, in which experiments exposure to hydroxyurea completely stops DNA replication for a few hours, then removing hydroxyurea, and measuring re-start of DNA replication by Polδ and Polε [35]. However, the relevance of such studies to most anti-cancer therapies remains unclear. The very selective mechanism of cytotoxicity of Ara-C, inhibition of DNA synthesis extension from Ara-CMP at the 3′ end of primers, causes a form of replication stress that is distinctly different from that caused by hydroxyurea, MMS or FTD (Figure 4). Thus, Ara-C provides a novel method for examining molecular mechanisms underlying cellular response to this new type of replication stress in future.

A critical question is what percentage of Ara-CMP is eliminated by the proofreading nuclease from the 3′ end of primers in the human cells. Biochemical studies with intact Polε could not address this question due to very strong intrinsic exonuclease activity associated with Polε. The current study indicated that IC_50_ dose of Ara-C is 2.7 and 15 nM for *POLE1^exo−/−^* and *wild-type* TK6 cells, respectively (Figure 5A). Around five times difference in the IC_50_ dose suggests that the proofreading nuclease eliminates 80% of the incorporated Ara-CMP. The proofreading nuclease of Polε (WT) does not discriminate Ara-CMP from dCMP incorporated at the 3′ end of primers (Figure 2C). Thus, a delay in the DNA synthesis extension from Ara-CMP, but not stronger affinity to Ara-CMP than dCMP, may cause nucleolytic elimination of Ara-CMP from the 3′ end of primers. We therefore proposed the model that the delayed extension causes replication stress as well as the elimination of incorporated Ara-CMP (Figure 7).

We investigated the role played by Polε exonuclease in response to three clinically used anti-viral nucleosides, abacavir (ABC), azidothymidine (AZT), and lamivudine, and showed that the proofreading exonuclease of Polε significantly contributes to cellular tolerance to ABC, AZT and lamivudine (Figures 4 and 5A). We also showed that a fraction of AZT is incorporated into the genomic DNA despite of its chain-terminating activity, and incorporated AZT interferes with the following round of DNA replication. It remains elusive how chain-terminating AZT is incorporated in the genomic DNA. Another unsolved question is the contribution of Polδ proofreading activity to cellular tolerance to nucleoside analogs. We postulate that Polδ proofreading activity is less important than Polε proofreading activity because blockage of Polδ-dependent lagging-strand DNA synthesis would not inhibit the progression of replication forks.

The current study reveals the selective sensitivity of the proofreading-nuclease-deficient cells to Ara-C. The data are useful for predicting the efficacy of Ara-C in individual malignant tumors, as heterozygous mutations in the exonuclease domain of Polε account for ~10% of colorectal cancer and endometrial cancer [36, 37]. In conclusion, isogenic mutants of DNA damage tolerance pathways are extremely useful for dissecting molecular mechanism underlying genotoxicity of nucleoside analogs.

## MATERIALS AND METHODS

### DT40 and TK6 cell culture

Culture conditions for DT40 cells, cell counting and cell cycle analysis have been described previously [11]. TK6 cells were cultured in an RPMI 1640 medium (Nacalai Tesque, Kyoto, Japan) supplemented with 10% heat-inactivated horse serum (HS) (GIBCO, lot No. 2017-06), 0.1mM Sodium pyruvate, L-glutamine (Nacalai Tesque), 50 U/mL penicillin, and 50 μg/mL streptomycin (Nacalai Tesque). The DT40 and TK6 cells were maintained at 39.5°C and 37°C respectively under a humidified atmosphere and 5% CO_2_.

### Plasmids

We used pX330 vector [38] (Addgene, US) for CRISPR Cas9 system [38, 39] and maker genes *DT-ApA*/*NEO^R^* (provided from the Laboratory for Animal Resources and Genetic Engineering, Center for Developmental Biology, RIKEN Kobe, http://www.cdb.riken.jp/arg/cassette.html) and *DT-ApA*/*PURO^R^* digested with *Apa*I and *Afl*II [40].

### Measurement of cellular sensitivity to DNA damaging agents

Methylcellulose colony formation assay was used for measuring the sensitivity of DT40 cells and TK6 cells to Ara-C, ABC, AZT, lamivudine, FTD and 5-FU as described previously [41]. In liquid-culture cell survival assay, DT40 cells were treated with DNA-damaging agents in 1 ml of medium using 24-well plates and incubated at 39.5°C for 48 hours. We transferred 100 μl of medium containing cells to 96-well plates and measured the amount of ATP using CellTiter-Glo (Promega), according to the manufacturer's instructions. Luminescence was measured by Fluoroskan Ascent FL (Thermo Fisher Scientific Inc, Whaltham, MA). To measure sensitivity, cells were treated with ICRF193 (Zenyaku Kogyo Company, Japan), camptothecin (Topogen, Inc, US), cisplatin (Nihonkayaku Inc, Japan), Olaparib (JS Research Chemicals Trading, Germany), Methl Methansesulfonate (MMS) (Nacalai tesque, Japan), ABC (Carbosynth, UK), Ara-C (Sigma, USA), Lamivudine (Wako, Japan), AZT (Sigma, USA), FTD (Wako laboratory chemicals, Japan), 5-FU (Nacalai tesque, Japan) and irradiated with ultraviolet (UV), and ionizing radiation (IR) (^137^Cs). To evaluate the relative cellular sensitivity of each mutant to *wild-type* cells, sensitivity curves were drawn by setting the survival of untreated cells as 100%. The concentration of 50% viability (inhibition concentration 50%; IC_50_) was determined from the sensitivity curves. The values of the mutant and *wild-type* cell lines were converted to a logarithmic scale (base 2). Each value was plotted on a bar graph.

### Generation of polymerase ε proofreading-exonuclease deficient DT40 cells

To mutate a conserved residue, Asp269, in the exonuclease catalytic site into Ala, we generated a *POLE1 exo^−^* mutation knock-in construct carrying a *BSR^R^* selection-marker cassette. Genomic DNA sequences in the *POLE1* (the catalytic subunit) gene were amplified using primers, 5′- CCTGTCTCCATGGCTGCAGACAGC -3′ and 5′- GCCAGGAGATGTCACTTCTGTCTC -3′ for the 5′-arm and 5′-CCCAGTTTCGTGGCTGCAGCATG-3′ and 5′- GGAGCGCGACCAGGCCAATGATGT -3′ for the 3′-arm of the knock-in construct. The resulting 1.8 kb 5′-arm and 4.3 kb 3′-arm were cloned into the pCR-TOPO BluntII vector (Invitrogen, CA). Point mutations for inserting the D269A amino acid replacement was introduced into the 5′ arm sequence using the primer, 5′- TGGGACAGTTTCCAGCTTCGCAAT -3′ and 5′- CCGTGTTCCAATTTGTGCCCGTTG -3′. The mutations create an additional *Tsp509*I site. The mutated 5′ arm and 3′ arm was ligated into the pBluescript vector. The *BSR^R^* selection-marker genes flanked by loxP sequences were inserted into the *Bam*HI site to generate *POLE1*-*exo^−^*-*BSR^R^*. To generate *POLE1^exo−/−^* cells, *wild-type* DT40 cells were transfected with *POLE1*-*exo^−^*-*BSR^R^*. The 0.5 kb genomic fragment was amplified using the primers, 5′- ATCTGTAAGGGAAATTGAGATGATG -3′ and 5′-TATTGAGACTCAATAAATGCAGCTC -3′, and used as a probe for Southern blot analysis to screen gene-targeting events. The *BSR^R^* selection-marker gene was removed by the transient expression of the CRE recombinase. Knock-in of the mutation was confirmed by digestion of the RT-PCR products with *Tsp509*I. The RT-PCR was conducted using following primers: 5′-CTGGTACAACGTGCGGTACCGCGGCAGC-3′ and 5′- CTGGTCCGTCTCTGGATCAGGAAACTTC-3′. The resultant *POLE1^exo-/+^* cells were transfected with *POLE1*-*exo^−^*-*BSR^R^* to make *POLE1^exo−/−^* cells.

### Generation of polymerase ε exonuclease- deficient TK6 cells

To mutate a conserved residue, Asp275, in the exonuclease catalytic site into Ala, we generated targeting construct from a genomic sequence covering the Polε p261(catalytic subunit) gene. *POLE1*-*exo^−^* mutation knock-in constructs (*POLE1-NEO^R^* and *-PURO^R^*) were generated from genomic PCR products combined with a resistance (*PURO^R^* and *NEO^R^*) gene cassette flanked by loxP signals at both ends (Supplementary Figure 6). The primers used to amplify the left arm were 5′- GCGAATTGGGTACCGGGCCTACACTGAATTTTCTCCTGT -3′ and 5′-CTGGGCTCGAGGGGGGGCCAGAGATGATATCTTCATTTC-3′, and the primers for the right arm were 5′-TGGGAAGCTTGTCGACTTAATGGCTTTATGCTTATTTTGT-3′ and 5′- CACTAGTAGGCGCGCCTTAACAAATGCTGCCCAGTTACTC-3′. The amplified fragments of left and right arms were assembled by seamless reaction (Invitrogen, US) into *DT-ApA*/*NEO^R^* (provided from the Laboratory for Animal Resources and Genetic Engineering, Center for Developmental Biology, RIKEN Kobe, http://www.cdb.riken.jp/arg/cassette.html) and *DT-ApA*/*PURO^R^* digested with *Apa*I and *Afl*II. The single and double underlines above indicate the homology of upstream and downstream from *Apa*I and *Afl*II sites respectively. The point mutations in right arm sequence resulting in D275A amino acid replacement was introduced by polymerase chain reaction (PCR) using following primers; 5′- GGTCGTCTCGATCGCAAATGCCAAAACCACAGGGTCCTG -3′ and 5′- TTGGCATTTGCGATCGAGACGACCAAACTGCCCCTCA AG-3′. The mutations create additional *Pvu*I site. We used pX330 vector (Addgene, US) for CRISPR Cas9 system. It is designed to recognize following sequence 5′- AAGAGTATCACGACTCCCTATGG-3′ for *POLE1-CRISPR1*, and 5′- GGTGTTCAGGGA GGCCTAATGGG-3′ for *POLE1-CRISPR2*. To generate *POLE1^exo−/−^* cells, *wild-type* TK6 cells were transfected with 2 μg each of targeting vectors (*POLE1-NEO^R^* and *POLE1-PURO^R^*) and 6 μg of the guide sequence-containing pX330 vector using NEON Transfection System (Life Technologies) at 1350 V, 10 msec, 3 pulses according to the manufacture's instructions. After 48 hours, the cells were plated in 96-well plates, and then subjected to puromycin (0.5 μg/ml) and neomycin (1 mg/ml). The drug-resistant cell colonies were picked on days 7-10 after transfection. The selection-marker gene was removed by the transient expression of CRE recombinase. Knock-in of the mutation was confirmed by digestion of the RT-PCR products with *Pvu*I.

### Generation of RAD18 deficient mutant TK6 cells

*RAD18* gene disruption constructs for TK6 cells, *RAD18-HYG^R^* and *RAD18-PURO^R^* were generated from genomic PCR products combined with *HYG^R^* and *PURO^R^* selection marker genes (Supplementary Figure 7). Genomic DNA sequences were amplified using the following primers: 5′- GCGAATTGGGTACCGGGCCGTTAATACAGCATAA -3′ and 5′- CTGGGCTCGAGGGGGGGCCTTGGGCAGCGGCTTC -3′ plus 5′- TGGGAAGCTTGTCGACTTAATAAATCAGGTAAAGTAAT -3′ and 5′- CACTAGTAGGCGCGCCTTAAAGCAACAAAAATGAA -3′ for the left arm and right arm, respectively. Left arm and right arm was inserted into *Apa*I and *Afl*II site of *DT-ApA*/*HYG^R^*, respectively, to create *RAD18-HYG^R^* using GENEART Seamless Cloning (Life Technologies). The single and double underlines above indicate the homology of upstream and downstream from *Apa*I and *Afl*II sites respectively. Similar to *RAD18-HYG^R^*, *RAD18-PURO^R^* was generated using *DT-ApA*/*PURO^R^*. *RAD18^−/−^* TK6 cells were generated using CRISPR Cas9 system. Briefly, guide sequences 5′- GAGCATGGATTATCTATTCA-3′ was inserted into the pX330 vector. TK6 cells were transfected with 2 μg each of targeting vectors (*RAD18-HYG^R^* and *RAD18-PURO^R^*) and 6 μg of the guide sequence-containing pX330 vector using NEON Transfection System (Life Technologies) according to the manufacture's instructions. After 48 hours, the cells were plated in 96-well plates, and then subjected to puromycin (0.5 μg/ml) and hygromycin (0.3 mg/ml). The drug-resistant cell colonies were picked on days 7-10 after transfection. The loss of *RAD18* transcript was confirmed by RT-PCR using primers 5′- AAGGAAATAAACAACAGCTCATTAAAAGGC-3′ and 5′- ATATCAATACAGCTAGAAGAATCCTCTTCT-3′. *GAPDH* transcripts were analyzed as a positive control for the RT-PCR analysis using primers 5′- TGGCCAAGGTCATCCATGACAACTT-3′ and 5′- GCGCCAGTAGAGGCAGGGATGATGT -3′.

### Immunofluorescence staining

Following treatment with 30 nM Ara-C, 100 nM FTD, or 10 μM 5-FU for 6 hours at 37°C, cells were incubated for 15 hours in drug-free medium at 37°C. Cells were collected on a glass slide using Cytospin (Shandon, Pittsburgh, PA, USA). Cells were fixed with 4% formaldehyde for 10 min at room temperature, permeabilized with 0.5% TritonX-100 and, after two rinses in PBS, were blocked in PBS in 5% bovine serum albumin (BSA). The cells were then incubated with anti-γH2AX(Ser139)MAb (Millipore) at a 1/1000 dilution in 5% BSA in PBS for 1 hour at room temperature. After three washes in PBS, the cells were incubated in Alexa Fluor 488 goat anti-mouse IgG antibody (Invitrogen) at a 1/1000 dilution in 5% BSA in PBS for 1 hour at room temperature, and after they were rinsed in PBS three times, cells were counterstained with 4′, 6′-diamidino-2-phenylindole (DAPI) and mounted with Vectashield (Vecta Laboratories). Cells were observed by the confocal laser scanning microscopy (TCS SP8, Leica Microsystems, Germany). At least fifty cells were scored per data point.

### Cell-cycle analysis

Cells were labeled for 15 min with 50 μM BrdU and chased with BrdU-free medium containing either zero or 30 nM Ara-C for eight hours. They were then harvested and fixed at 4°C overnight with 70% ethanol, and successively incubated as follows: (i) in 2N HCl, 0.5% Triton X-100 for 30 min at room temperature; (ii) in FITC-conjugated anti-BrdU antibody (Becton, Dickinson and Company, Franklin Lakes, NJ) for 30 min at room temperature; (iii) in FITC- conjugated anti - mouse antibody (Southern Biotech, Birmingham, AL) for 30 min at room temperature; (iv) in 5 μg/ml PI in PBS. Subsequent flow cytometric analysis was performed on an LSRFortessa (Becton, Dickinson and Company). Fluorescence data were displayed as dot plots using the Cell Quest software (Becton, Dickinson and Company).

### Polε holoenzyme protein purification

The human Polε holoenzyme, with N-terminal His-tagged p261 and N-terminal Flag-tagged p59, was expressed using a baculovirus vector (pBacPAK9, Clontech, Palo Alto, CA) in insect cells (High Five, Life Technologies, Palo Alto, CA). To inactivate proofreading exonuclease activity, p261 Asp275 was replaced by Ala in the p261 transgene. The Polε holoenzyme was obtained by the following four-step purification [24]: Polε was initially purified by the DEAE column. Polε was purified by the affinity of the Flag-tag using an anti-Flag tag affinity column and was subsequently purified the affinity of the His-tag using a Ni-resin column. Polε carrying a complete set of the four components was purified with the glycerol gradient. The concentrations and purity of the proteins were estimated from the intensity of protein bands in an SDS-polyacrylamide gel (Supplementary Figure 2).

### Primer extension assays

*In vitro* DNA synthesis analysis was carried out with 0.06 pmol ^32^P-labeled primer in a reaction mixture (5 μl) containing 30 mM HEPES-NaOH (pH 7.4), 7 mM MgCl_2_, 8 mM NaCl, 0.5 mM dithiothreitol, and 0 μM or 10 μM each dNTP in the presence of Polε for 15 min at 37°C. At the end of the reaction, the products were denatured with formamide and loaded onto 15.6% polyacrylamide gels containing 7 M urea in TBE buffer (89 mM Tris, 89 mM boric acid, 2 mM EDTA). After electrophoresis, radioactivity was measured with a Fuji Image analyzer, FLA2500 (Fujifilm, Tokyo, Japan). Reactions for Figure 3 were carried out with either 40 nM Polε (WT) or 2.5 nM Polε (exo-) and 8 nM of the primer/template substrate in a 5 μl reaction mixture containing various concentrations of Ara-CTP and 10 μM dNTP.

### Synthesis of nucleotides and oligo-nucleotides

The oligonucleotides, d(TCCGTTGAAGCCTGC TTT)X, where X represents carbovir, or lamivudine, were chemically synthesized as described previously [42]. The 3′ Ara-C docking oligo was previously used [31]. The 5′-triphosphate of Ara-C was synthesized from 1-(β-D-arabinofuranosyl)cytosine according to the previous method with a slight modification [43]. The 5′-triphosphates of carbovir and lamivudine were synthesized according to a previously published method, with a slight modification [44], followed by deprotection by treatment with 28% ammonia water at 55°C for 5 h. The crude reaction mixtures were loaded on a column (1.6 × 27 cm) containing DEAE-cellulose resins (Wako Pure Chemical Industries, Ltd., Osaka, Japan), and the triphosphate derivatives were purified with a linear gradient of 0–0.5 M triethylammonium bicarbonate buffer (pH 8.0). The aimed products were eluted at 0.4–0.5 M buffer, and the pooled fractions were evaporated to dryness. The purified Ara-C, carbovir, and lamivudine triphosphates were analyzed by mass spectrometry, and their *m/z* values were found to be 482.2 ([M–H]^−^, *m/z* 482.1 calcd for C_9_H_15_N_3_O_14_P_3_), 486.3 ([M–H]^−^, *m/z* 486.2 calcd for C_11_H_15_N_5_O_11_P_3_), and 468.1 ([M–H]^−^, *m/z* 468.2 calcd for C_8_H_13_N_3_O_12_P_3_S), respectively. A ^31^P NMR spectrum of lamivudine triphosphate was also measured (Supplementary Figure 3C).

## SUPPLEMENTARY MATERIALS FIGURES


