# Supplementary material for: The dominant role of proofreading exonuclease activity of replicative polymerase ε in cellular tolerance to cytarabine (Ara-C)
Source: Oncotarget. 2017 Mar 23;8(20):33457–74. doi: 10.18632/oncotarget.16508 (PMC5464882; doi:10.18632/oncotarget.16508)
Supplement: Supplementary file 1 [file oncotarget-08-33457-s001.pdf]

The dominant role of proofreading exonuclease activity of replicative polymerase ε in cellular tolerance to cytarabine (Ara-C)

SUPPLEMENTARY MATERIALS

SUPPLEMENTARY FIGURES

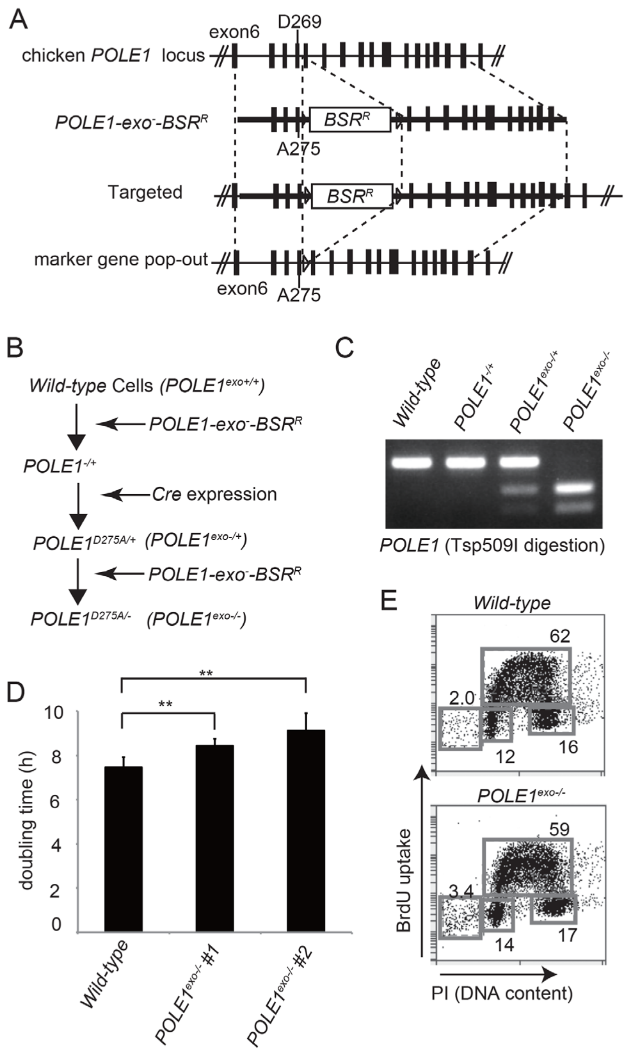

Supplementary Figure 1: Inactivation of Polε exonuclease in DT40 cells. **(A)** Schematic representation of the *POLE1* locus in DT40 cells and the structure of the gene-targeting constructs. The close solid boxes indicate the regions of exons. Triangles indicate lox^P sequences. BSR^R designates the blasticdin resistant gene expression cassette. **(B)** The targeting strategy of *POLE1* locus. **(C)** The RT-PCR product amplified from the D275A mutant mRNA, but not *wild-type* mRNA, was digested with the *Tsp*509I restriction-enzyme. Note that silent mutations that generate a *Tsp*509I site were introduced near the D275A mutations. **(D)** The average doubling time for the indicated genotypes. Error bars show the SD in at least three independent experiments. Statistical significance (by Student's *t*-test) is as follows: ** $P<0.01$. **(E)** Representative cell-cycle distribution for the indicated genotypes. The top of the box, and the lower left, lower right, and left-most gates correspond to cells in the S, G_1, and G_2/M phases, and the sub-G_1 fraction, respectively. The sub-G_1 fraction represents dying and dead cells. The percentage of cells in each gate is indicated.

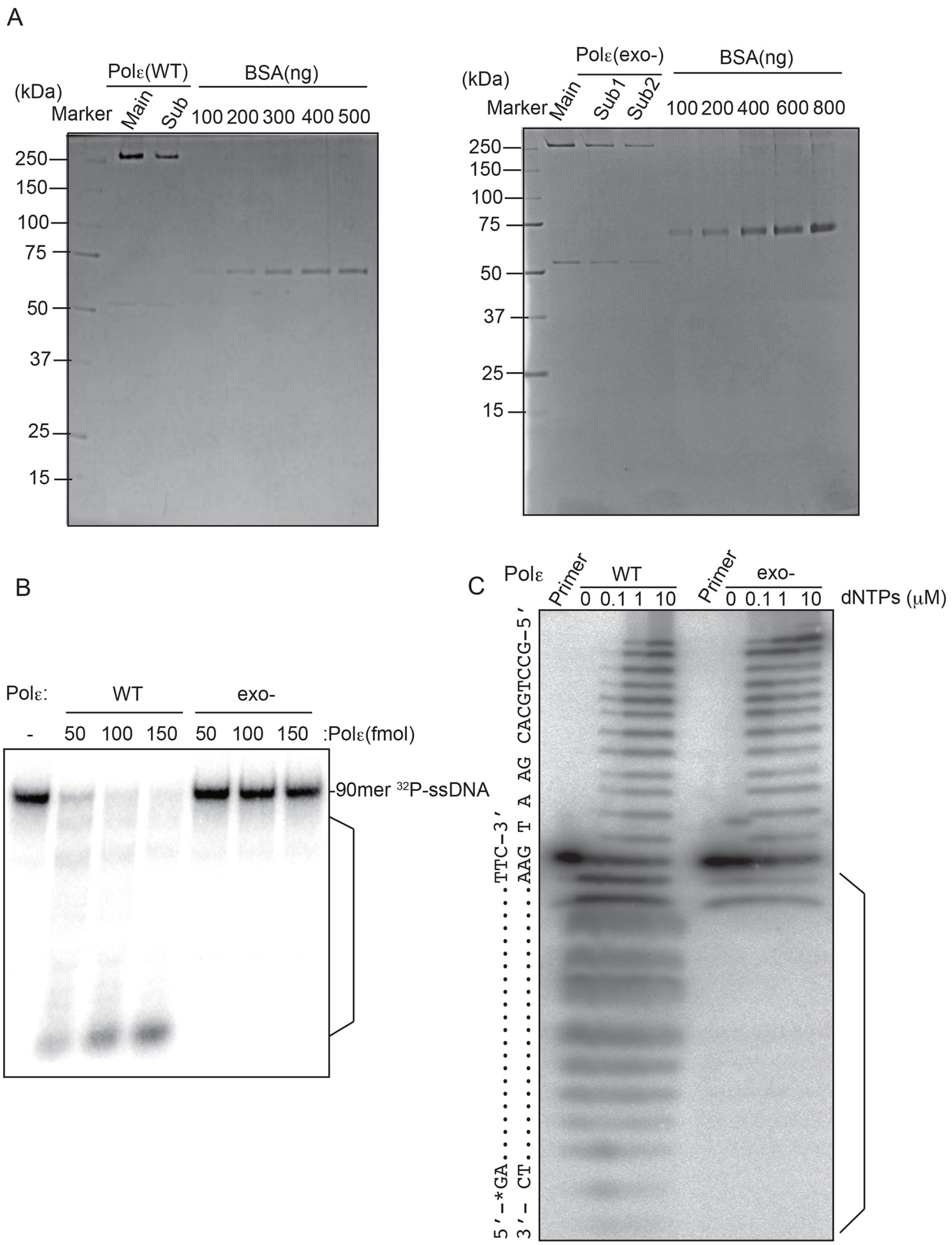

Supplementary Figure 2: Expression and purification of recombinant human Polε(WT) holoenzyme and mutant Polε(exo-) holoenzymes. **(A)** The Polε holoenzymes were obtained in four-step purification as described in materials and methods. Concentrations and purities of purified proteins were estimated from intensities of the protein bands in an SDS-polyacrylamide gel using bovine-serum albumin as a standard. **(B)** The exonuclease activity of purified Polε(WT) and Polε(exo-) holoenzymes. 90mer single-stranded DNA having ^{32}P at the 5' end was incubated with the indicated amount of the enzymes for 15 min in the absence of dNTPs. The open parenthesis represents the position of degraded products. **(C)** DNA synthesis activity of purified Polε(WT) and Polε(exo-) holoenzymes. Reaction was carried out with 40 nM Polε and 8 nM of the primer/template strands with indicated concentration of dNTPs. Used primer/template was shown in left. The position of radiolabel with ^{32}P is noted with asterisk. The open parenthesis represents the position of degraded products.

A

```
5′-*TCCGTTGAAGCCTGCTTTG-3′
3′- AGGCAACTTCGGACGAAACGAGCACGTCCG-5′

5′-*TCCGTTGAAGCCTGCTTTA-3′
3′- AGGCAACTTCGGACGAAATCAGCACGTCCG-5′
```

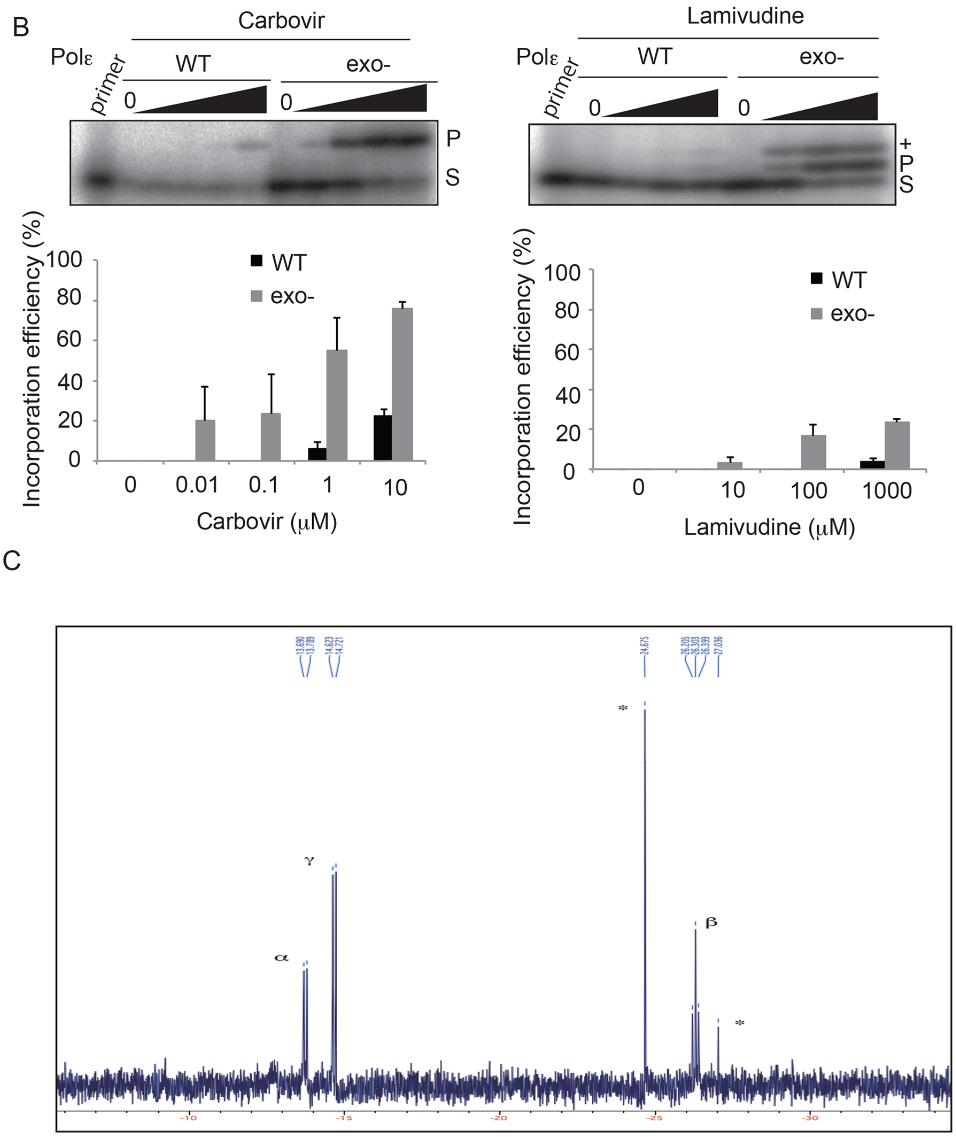

Supplementary Figure 3: Polε incorporates nucleotide analogs *in vitro*. **(A)** Sequences of oligonucleotide primers and templates. The upper and lower primer and template are used for the analyses with lamivudine triphosphate and carbovir (a triphosphated form of ABC) respectively. The position of radiolabel with ^{32}P is noted with asterisk. **(B)** A single nucleotide insertion by Polε (WT) and Polε (exo-) with varying concentrations of the indicated nucleotide analogs. The actual concentrations are shown in lower panel. Reaction was carried out with 40 nM Polε and 8 nM of the primer/template strands in the absence of dNTPs. The substrate 'S' means primers ^{32}P radiolabeled at the 5' end. 'P' represents product of the nucleoside analogs incorporation. '+' represents the product incorporating a contaminant described in **(C)**. The relative yield of products is plotted against increasing nucleoside analog concentration as mean ±SD of three independent experiments. **(C)** ^{31}P NMR of Lamivudine-triphosphate. Purified lamivudine-triphosphate was analyzed by ^{31}P NMR in D_2O with an external standard of trimethylphosphate referenced at 0 ppm. Two doublet signals at –13.74 and –14.67 ppm and a triplet signal at –26.30 ppm were assigned as α-, γ-, and β-phosphate resonances of lamivudine-triphosphate, respectively. The signals marked with an asterisk seemed to be impurities.

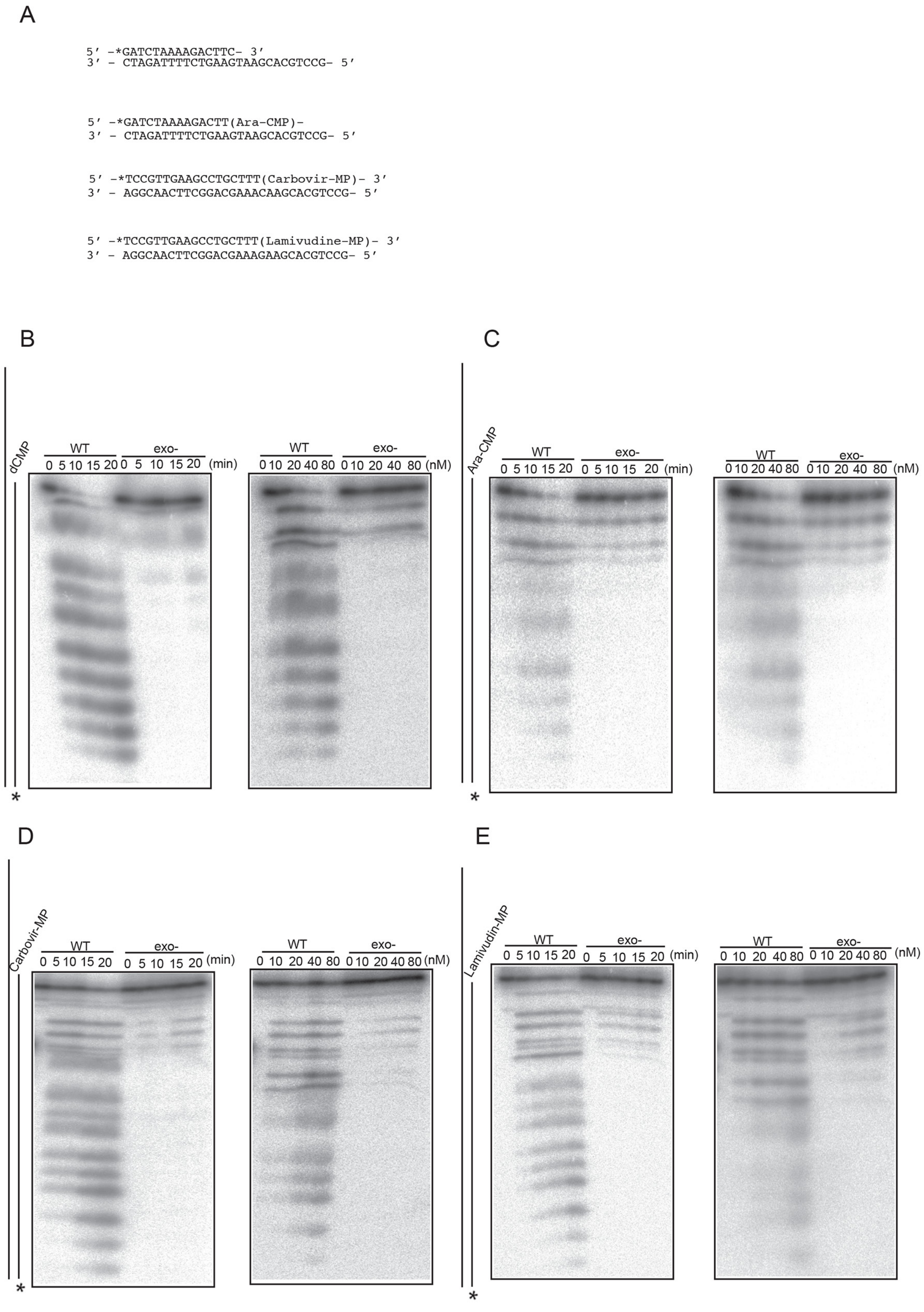

Supplementary Figure 4: Polε is able to remove CTNAs from the 3' end of primers *in vitro*. **(A)** The nucleotide sequences of primers and template sequences. The position of radiolabel with ^{32}P is noted with asterisk. **(B-E)** Kinetics of the primer degradation by Polε(WT) holoenzyme and Polε(exo-) holoenzyme. The experiments were done in the absence of dNTPs. In the left panels of **(B-E)**, reactions were carried out with 40 nM of Polε(WT) and Polε(exo-) holoenzymes for the indicated duration. In the right panels, reactions were carried out with the indicated concentrations of Polε(WT) and Polε(exo-) holoenzymes for 15 min. The amount of the intact primer does not change by any incubation with Polε(exo-) holoenzyme, indicating that degraded products are not attributable to contaminated nuclease.

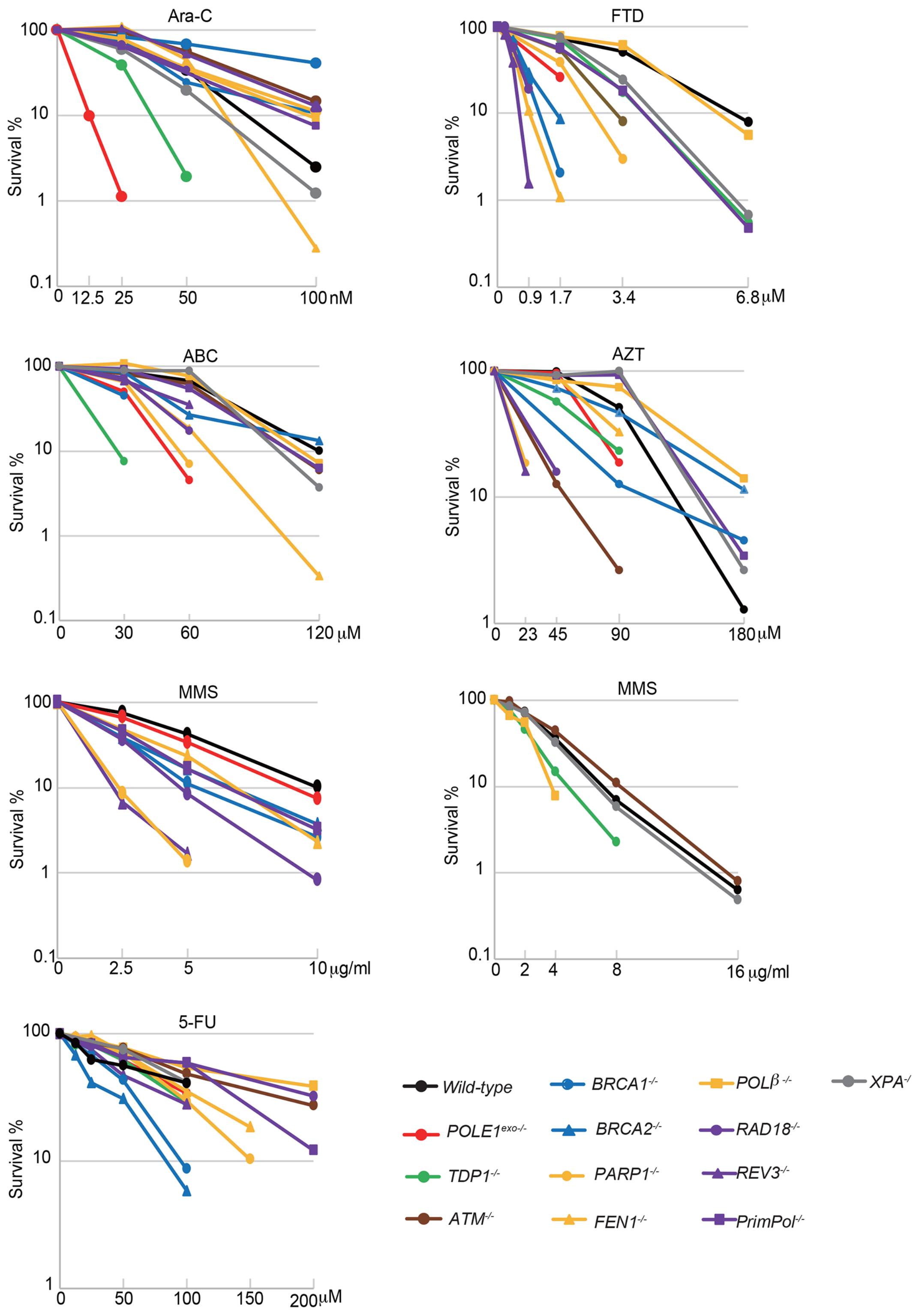

Supplementary Figure 5: Sensitivity profiles of the indicated nucleoside analogs and MMS in isogenic DT40 mutants. Survival curve of cells treated with the indicated nucleoside analogs. The sensitivity of cells to these nucleoside analogs was measured with methylcellulose colony formation assay. The sensitivity of cells to MMS was measured with liquid-culture cell survival assay. The dose is displayed on the x-axis on a linear scale, while the percentage fraction of surviving cells is displayed on the y-axis on a logarithmic scale. Error bars show the SD of mean for three independent assays. The values of each IC_{50} were calculated as the concentration, at which the colony survival was decreased to 50% of untreated cells.

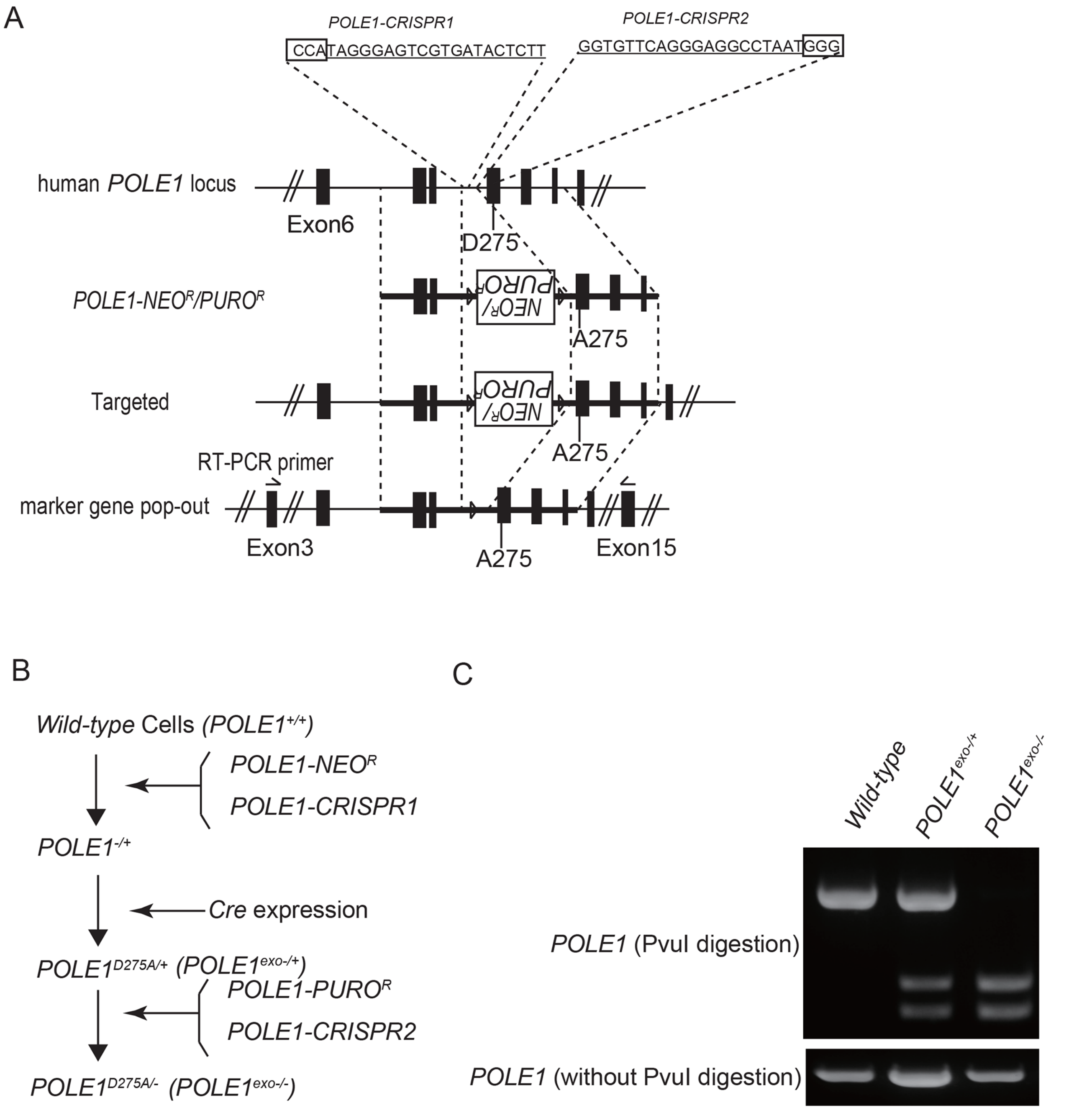

Supplementary Figure 6: Inactivation of Polε exonuclease in TK6 cells. (A) Schematic representation of the *POLE1* locus in TK6 cells and the structure of the gene-targeting constructs. The close solid boxes indicate the coding regions of exons. Triangles indicate lox^{P} sequences. *PUPO*^{R} and *NEO*^{R} designate the puromycin and reomycin resistant gene expression cassette respectively. **(B)** The targeting strategy of *POLE1* locus. **(C)** The RT-PCR product amplified from the D275A mutant mRNA, but not *wild-type* mRNA, was digested with *Pvu*I. The silent mutation that generates *Pvu*I site was introduced nearby D275A mutation.

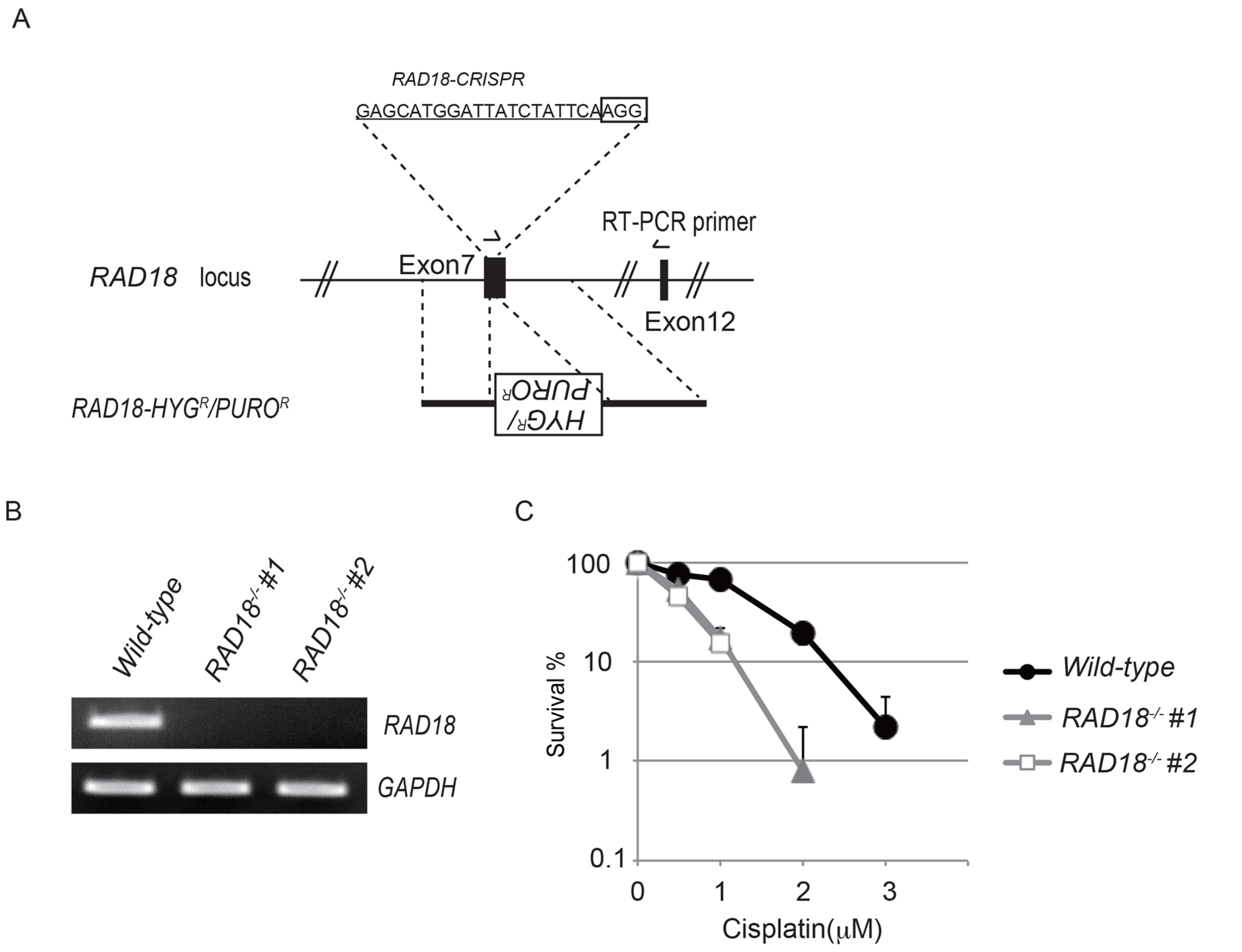

Supplementary Figure 7: Generation of *RAD18* knockout TK6 cell lines. **(A)** schematic of *RAD18*-gene-disruption and the sequence of guide RNA. Exon7 was replaced with antibiotics makers. Arrows indicate a set of primers for reverse transcription PCR (RT-PCR) designed at exon 7 and exon 12, respectively. *HYG*^{R} designates the hygromycin B resistant gene expression cassette. **(B)** RT-PCR analysis to confirm the gene disruption of *RAD18*. GAPDH is a loading control. **(C)** The sensitivity of *RAD18*$^{-/-}$ TK6 cells to cisplatin.

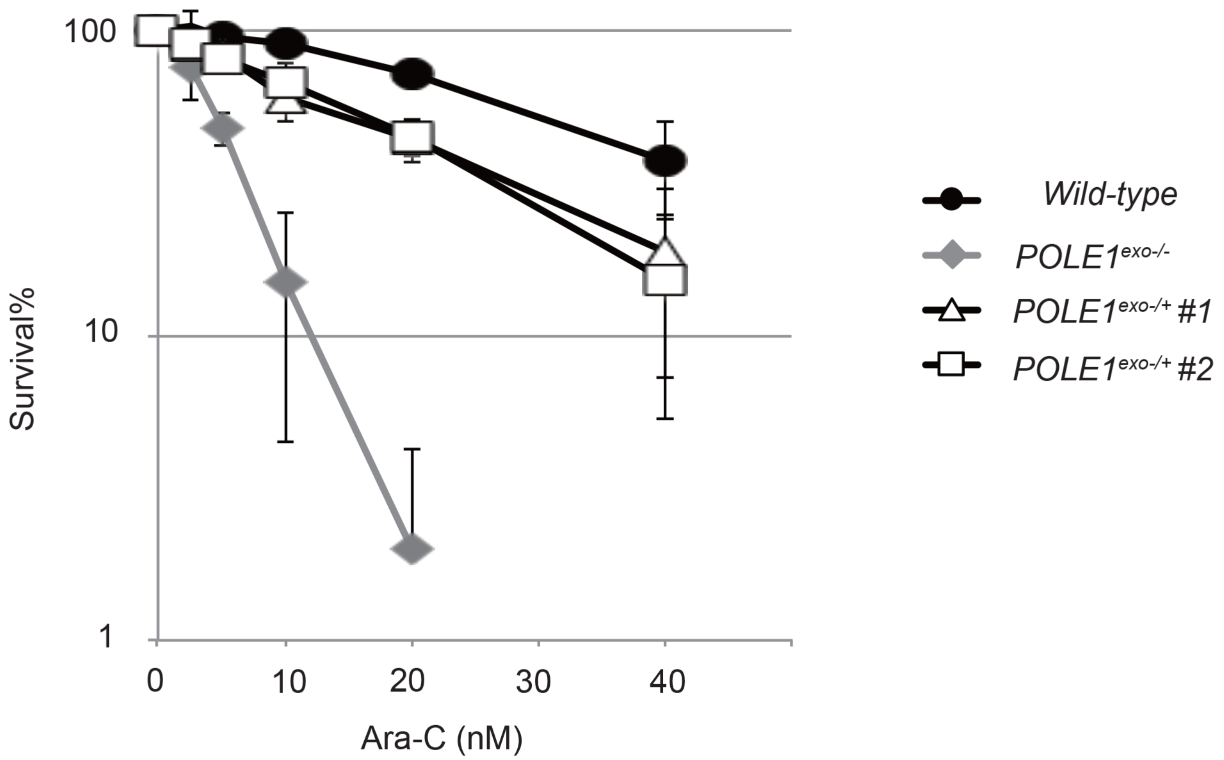

Supplementary Figure 8: The sensitivity of *POLE1*$^{exo-/-}$ TK6 cells to Ara-C. The fractions of surviving colonies relative to those of untreated controls are shown on the y-axis on a logarithmic scale, while the concentrations of the nucleoside analogs are displayed on the x-axis on a linear scale. Error bars show the SD of the mean for three independent assays.

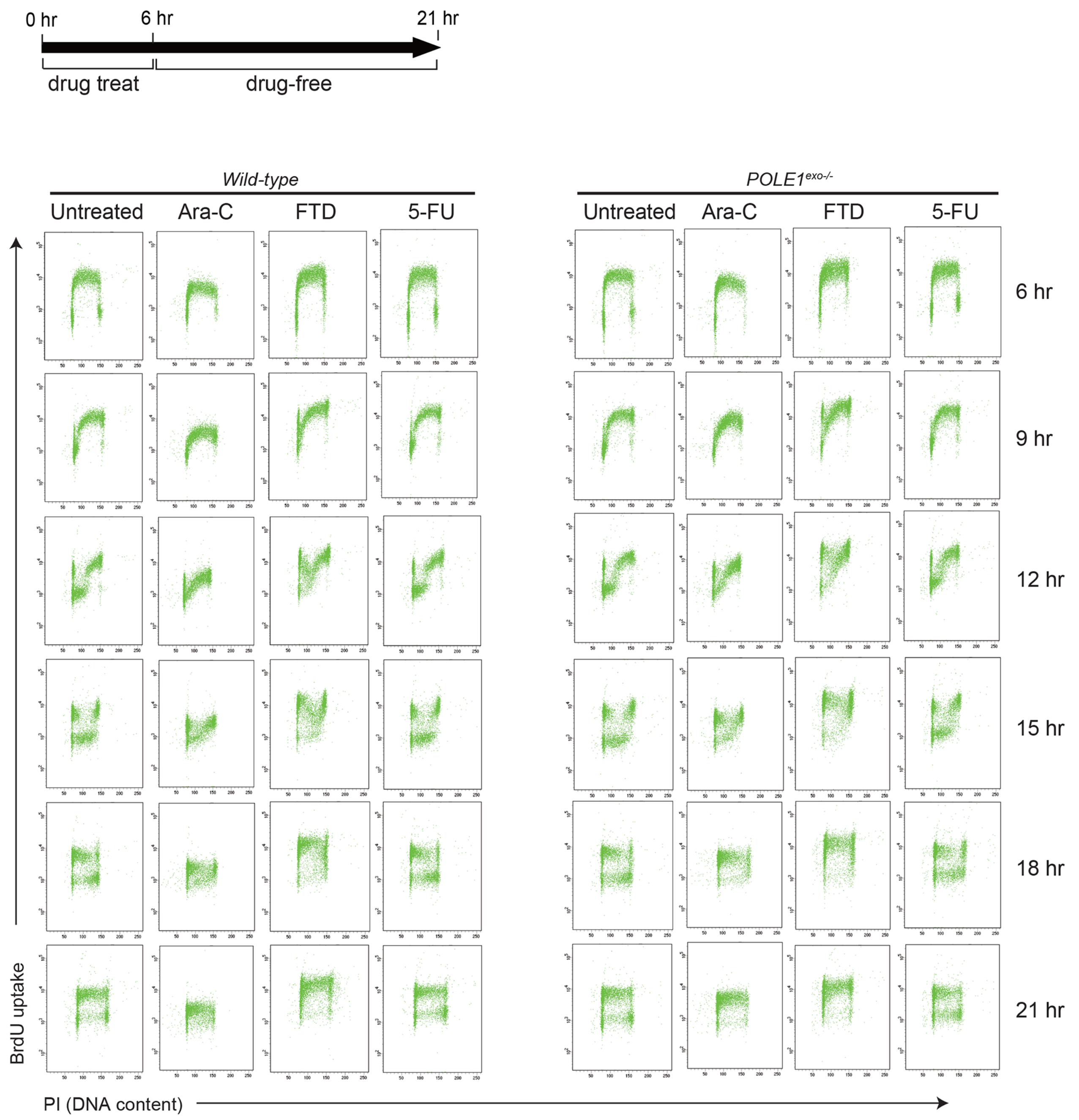

Supplementary Figure 9: Limited effect of Ara-C, FTD and 5-FU treatment on cell cycle progression. Following treatment with 30 nM Ara-C, 100 nM FTD or 10 µM 5-FU for 6 hour at 37°C, cells were pulse-labeled with BrdU for 15 min, and subsequently incubated in drug-free medium. Cell cultures were harvested at the indicated time.

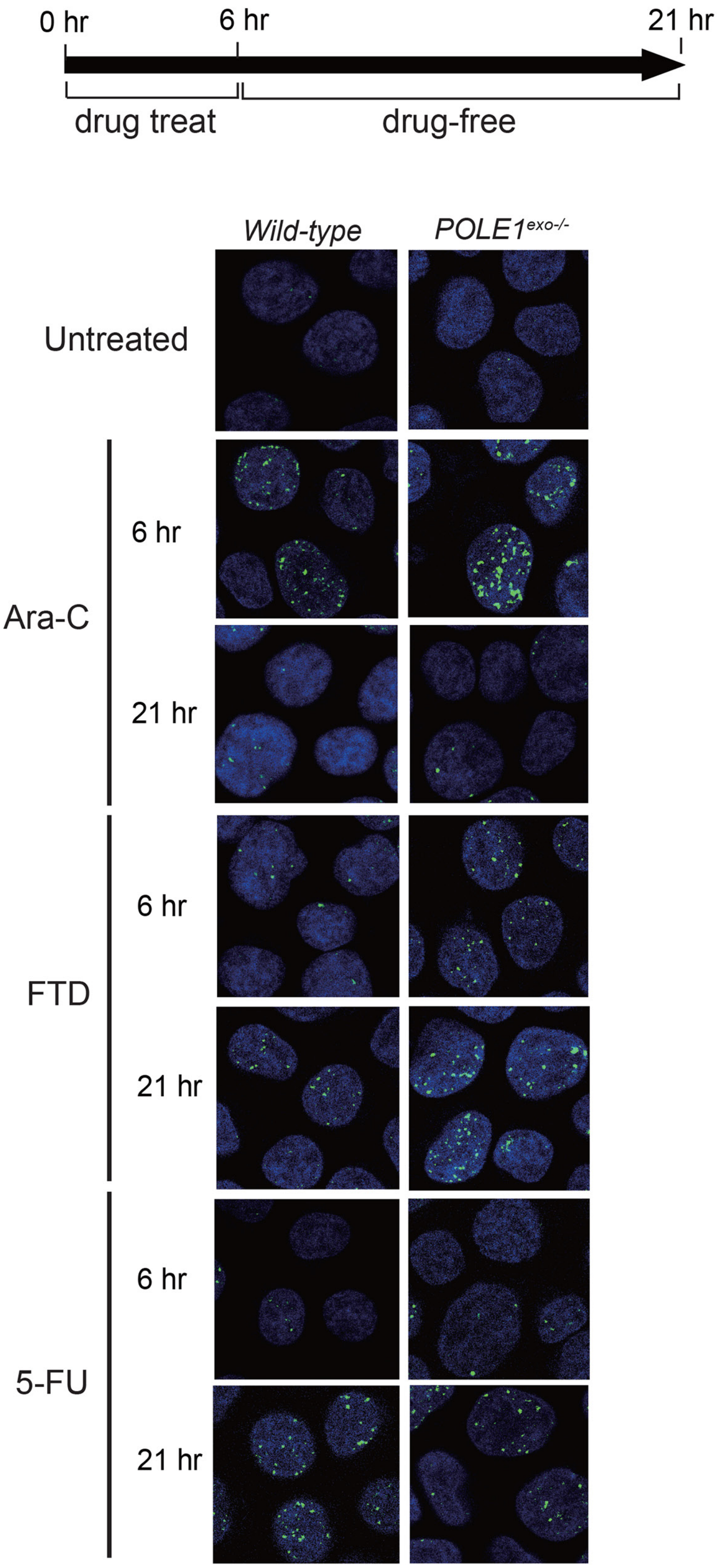

Supplementary Figure 10: Representative image of γH2AX subnuclear foci of indicated cells. Following pulse-treatment with 30 nM Ara-C, 100 nM FTD, or 10 µM 5-FU for 6 hours, cells were incubated for 15 hours in drug-free medium. γH2AX foci were measured at 6 and 21 hours.

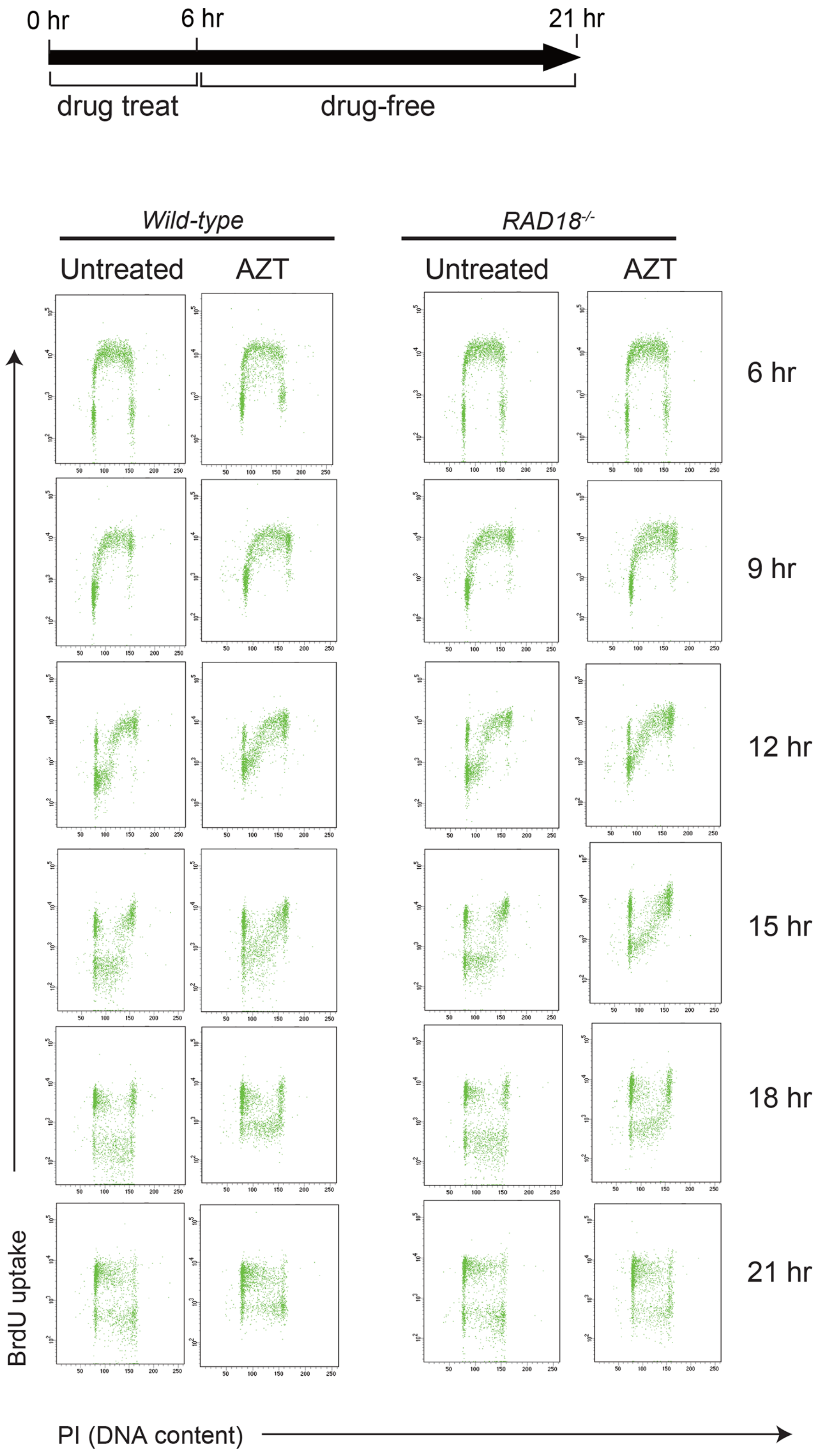

Supplementary Figure 11: Limited effect of AZT treatment on cell cycle progression. Following treatment with 100 µM AZT for 6 hour at 37°C, indicated TK6 cells were pulse-labeled with BrdU for 15 min, and subsequently incubated in drug-free medium. Cell cultures were harvested at the indicated time.

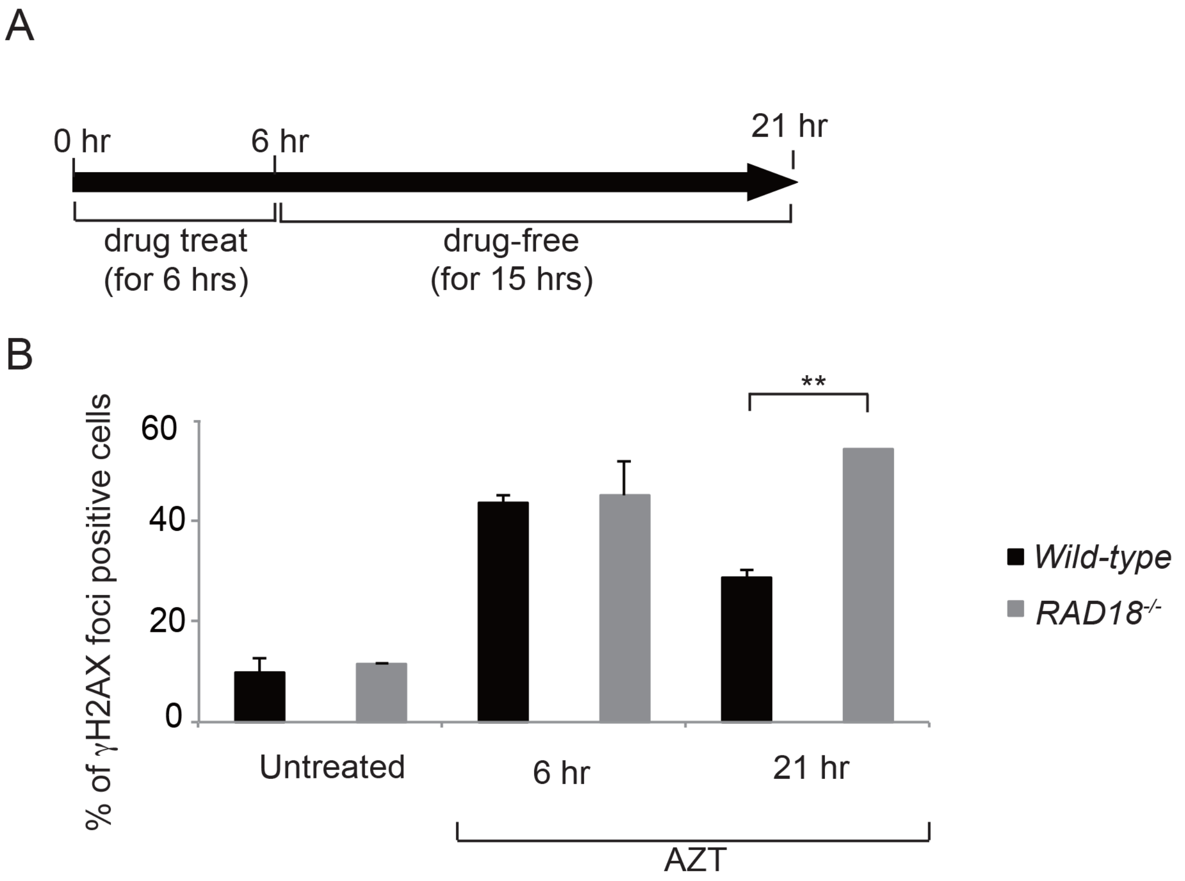

Supplementary Figure 12: (A) The experimental protocol for the immunofluorescent visualization of subnuclear γH2AX focus formation in *wild-type* and *RAD18*$^{-/-}$ TK6 cells. **(B)** Following pulse-treatment with 100 µM AZT for 6 hour at 37°C, cells were incubated for 15 hours in drug-free medium. γH2AX foci were measured at 6 and 21 hours. The bar graph represents mean and SD of % γH2AX-foci positive cells (> seven foci per cell) in three independent experiments. At least fifty nuclei were scored in each case. Statistical significance (by Student's *t*-test**)** is as follows: ** *P*<0.01.
